# Macroevolutionary decline in mycorrhizal colonization and chemical defense responsiveness to mycorrhization

**DOI:** 10.1016/j.isci.2023.106632

**Published:** 2023-04-10

**Authors:** Ludovico Formenti, Natalie Iwanycki Ahlstrand, Gustavo Hassemer, Gaëtan Glauser, Johan van den Hoogen, Nina Rønsted, Marcel van der Heijden, Thomas W. Crowther, Sergio Rasmann

**Affiliations:** 1 Laboratory of Functional Ecology, Institute of Biology, University of Neuchâtel, Neuchâtel, Switzerland; 2 Institute of Ecology and Evolution, Terrestrial ecology, University of Bern, Bern, Switzerland; 3 Natural History Museum of Denmark, University of Copenhagen, Øster Voldgade 5–7, 1350 Copenhagen, Denmark; 4 Neuchâtel Platform of Analytical Chemistry (NPAC), University of Neuchâtel, Neuchâtel, Switzerland; 5 Department of Environmental Systems Science, Institute of Integrative Biology, ETH Zürich, Zürich, Switzerland; 6 National Tropical Botanical Garden, Kalaheo, HI 96741, USA; 7 Plant-Soil Interactions, Institute for Sustainability Sciences, Agroscope, 8046 Zürich, Switzerland

**Keywords:** Microbiome, Plant ecology, Plant evolution

## Abstract

Arbuscular mycorrhizal fungi (AMF) have evolved associations with roots of 60% plant species, but the net benefit for plants vary broadly from mutualism to parasitism. Yet, we lack a general understanding of the evolutionary and ecological forces driving such variation. To this end, we conducted a comparative phylogenetic experiment with 24 species of *Plantago,* encompassing worldwide distribution, to address the effect of evolutionary history and environment on plant growth and chemical defenses in response to AMF colonization. We demonstrate that different species within one plant genus vary greatly in their ability to associate with AMF, and that AMF arbuscule colonization intensity decreases monotonically with increasing phylogenetic branch length, but not with concomitant changes in pedological and climatic conditions across species. Moreover, we demonstrate that species with the highest colonization levels are also those that change their defensive chemistry the least. We propose that the costs imposed by high AMF colonization in terms of reduced changes in secondary chemistry might drive the observed macroevolutionary decline in mycorrhization.

## Highlights

• AMF colonization decreases monotonically with increasing phylogenetic branch length
• AMF colonization is not related to macro-pedo-climatic conditions across species
• Production of defensive chemistry negatively correlates with AMF colonization
• Reduction in AMF colonization might be driven by energetic costs

## Graphical abstract

**Figure:**
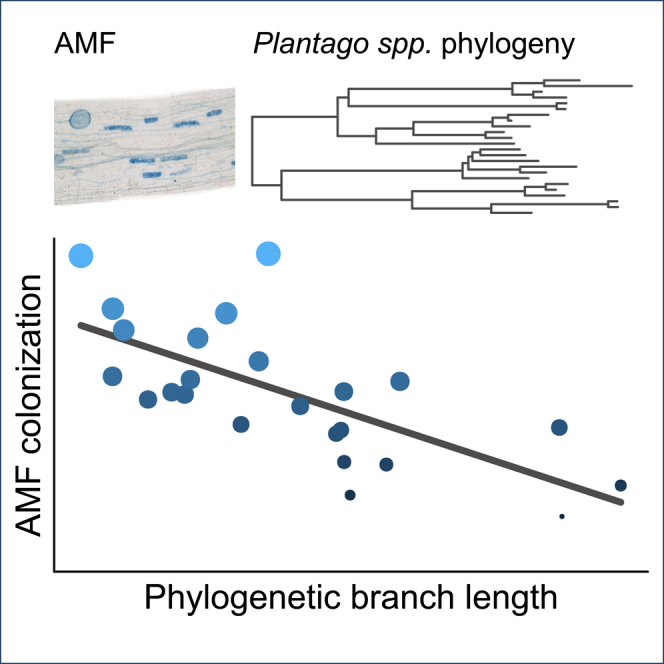

## Introduction

Mutualisms among species shape ecological communities and ecosystem dynamics worldwide.^1^^,^^2^^,^^3^ Among the most ancient and widespread mutualistic symbioses are the associations between plant roots and mycorrhizal fungi. Mycorrhizae acquire photosynthates directly from plants, and in exchange they expand the plants’ foraging capacities,^4^ therefore stimulating plant growth and resistance against abiotic and biotic stressors.^5^^,^^6^ In addition to facilitate nutrient acquisition, arbuscular mycorrhizal fungi (AMF), particularly, have been shown to alleviate plant stress caused by abiotic environmental conditions,^7^^,^^8^ and also biotic stress such as herbivory or pathogen attack.^6^ However, although widespread and putatively beneficial, plant-AMF interactions can vary from being highly mutualistic to parasitic. In this latter scenario, the mycorrhizae can inhibit plant growth by imposing strong energetic costs to the plants.^9^^,^^10^ To date, ecologists still struggle to understand the sources of variation in mycorrhization levels and their effects on plant phenotypes. Sources of variation include biotic factors such as the plants and fungal genetic make-up, or abiotic factors such as climatic conditions.^10^^,^^11^

If the association with AMF is a phylogenetically constrained trait, closely related species should sustain similar mycorrhization intensities, and respond similarly to mycorrhization. Although at higher taxonomic levels, such as across plant families, a phylogenetic constraint has been found for plant responsiveness to AMF colonization, the intensity of colonization and influence on ecosystem functioning,^2^^,^^12^ it remains unclear whether this selection exists at a finer taxonomic level, such as across species belonging to the same genus. On the other hand, distantly related plants inhabiting similar environments might favorably associate with components of the local fungal community, independent of their evolutionary history.^13^ Therefore, AMF colonization intensity and plant responsiveness to AMF colonization can be driven by either shared evolutionary history,^12^ or ecological-niche convergence because of shared abiotic and the biotic factors.^14^

Although patterns of plant responses to AMF are relatively well documented for plant growth or the allocation of biomass to different organs across species with different life histories,^10^^,^^15^ our understanding of how plants respond to AMF by changing their chemical phenotype, within biogeographical or phylogenetic variation, is largely lacking.^16^^,^^17^ Plants produce a plethora of molecules that are not directly linked to primary metabolism, referred to as secondary or specialized metabolites, that serve to mediate interactions with the biotic and abiotic environments.^18^ For instance, such specialized metabolites combine with plant physical defenses to shape the plant defensive phenotype against a wide range of herbivores and pathogens.^19^ AMF can modify plant specialized metabolite production in both roots and aboveground plant parts,^20^^,^^21^^,^^22^ in turn modifying plants’ interactions with herbivores, pathogens, and higher trophic levels.^23^^,^^24^ However, how plant evolutionary history and ecological factors modulate the effect of AMF on plant chemical defense traits remains uncertain.

**Figure 1:**
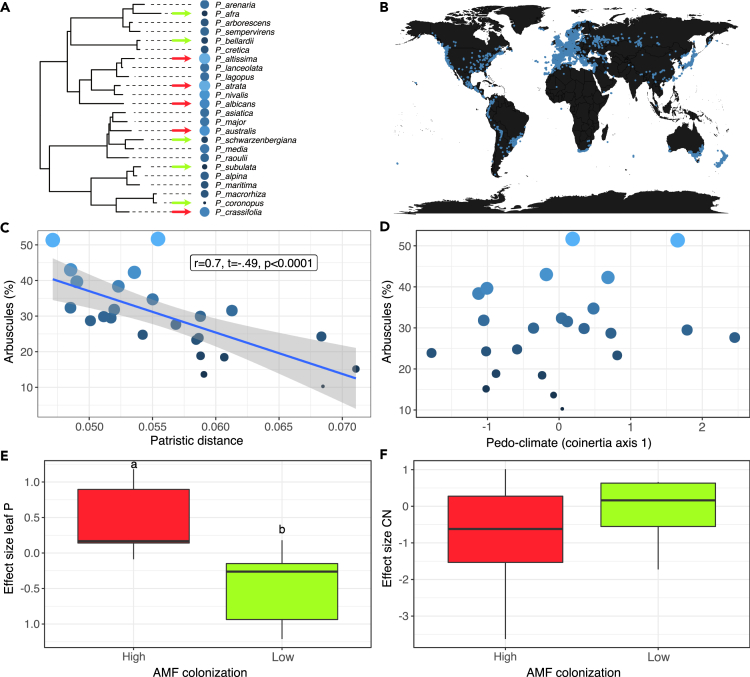
Phylogenetic and macroecological patterns in mycorrhization (A) The phylogenetic tree of the 24 species of *Plantago* is studied (see also Table S1). The tips of the phylogeny are color-coded based on the root-to-tip distance of each species (lighter colors indicate shorter branch length), see also Figure S5. The size of the dot on the tips of the phylogeny is proportional to the number of arbuscules (arbuscular mycorrhizal fungi (AMF) colonization intensity) of each of the 24 *Plantago* species. (B) represents the distribution around the globe of the 24 *Plantago* species used in the study. (C) and (D) represent the correlation between AMF colonization intensity phylogenetic branch length, and pedo-climatic niche distance, respectively. Dots are coded based on the root-to-tip distance of each species, with lighter colors indicating shorter branch length. Blue line and grey-shaded area around the line represent 95% confidence level interval for predictions from a linear model. (E) and (F) represent, for total leaf phosphorus (leaf P μg mg^−1^) and carbon to nitrogen ratio (CN % biomass), respectively, the average effect of AMF colonization intensity represented as the Cohen’s D effect size between mycorrhized and non-mycorrhized plants. Red boxes (High) show the average of five species that are highly mycorrhized (see red arrows in Figure 1A), whereas green boxes (Low) show the average of five low mycorrhized species (see green arrow in Figure 1A). Boxplots represent the minimum (black dots), first quartile (black line), median (bold black line), third quartile (black line), and maximum (black dots) of data distribution. Letters above boxes indicate significant difference between groups (PGLS; p < 0.05).

In this study, we question how phylogenetic history and pedo-climatic convergence affect AMF colonization intensity and responsiveness in relation to plant growth and chemical defense traits. To this end, we studied patterns of AMF colonization and plant responses across 24 species in the genus *Plantago* L. (Figure 1A), by growing them from seed in a common environment with and without inoculation of four common and widespread AMF species; *Rhizophagus irregularis*, *Funneliformis mosseae*, *Claroideoglomus claroideum*, and *Diversispora celata*. The genus *Plantago* is exceptionally well-studied phylogenetically and chemotaxonomically.^25^^,^^26^ A large fraction of the estimated 250 species in the genus have been shown to produce a diverse array of monoterpenoid derived iridoid glycosides (IGs), which are recognized as chemotaxonomic markers for *Plantago*.^25^ IGs are generally regarded to function as allelochemicals and are involved in plant defenses with antimicrobial and/or antiherbivore properties.^27^^,^^28^ Moreover, the genus *Plantago* is distributed worldwide and species are found in habitats ranging from desertic-mediterranean to temperate-continental climate niches.^29^ Because this range of habitats are associated with variable environments, herbivore and pathogen communities, ecological convergence may have shaped AMF-*Plantago* interactions, as well as growth patterns and specialized metabolites’ production, making the genus an excellent case for this investigation. By adopting a phylogenetic-multifunctional approach, we asked: (1) How are mycorrhizal colonization levels across species related to phylogenetic history or edaphic and climatic conditions? (2) How does AMF colonization affect plant growth and IG variation? (3) What is the relationship between colonization levels and AMF-mediated plant growth or chemical changes? First, we predicted that if AMF stimulate growth and defense traits, resource-poor and stressful environments would select for species with high investment in AMF colonization intensities. Second, because of evolutionary tendencies for family-wide losses in plant-AMF association, rather than recent gains, we predicted that over time, plant clades in the genus *Plantago* would tend to reduce their association with AMF. Finally, because AMF are known to stimulate plant growth and chemical defences,^30^ we predicted that species with low mycorrhization levels are also less plastic in responses to AMF colonization such that they exhibit reduced responsiveness to mycorrhization.

## Results

**Figure 2:**
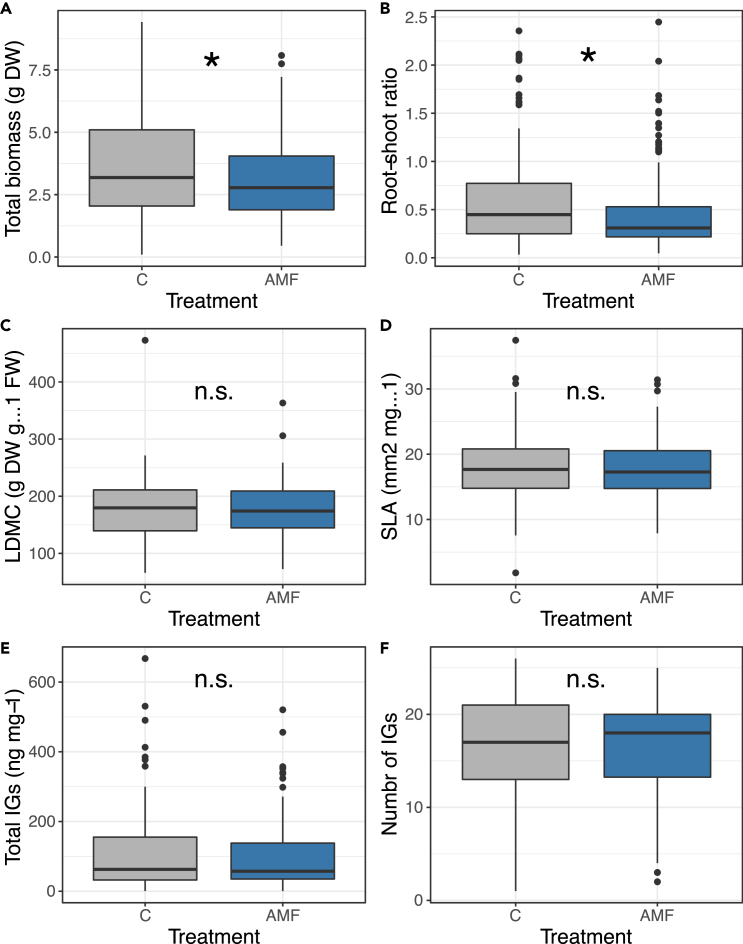
Effect of arbuscular mycorrhizal colonization of plant growth and chemical defense traits (A) shows the total plant biomass, (B) shows aboveground-belowground biomass allocation, (C) and (D) show plant leaf traits (leaf dry matter content LDMC, and specific leaf area SLA), whereas (E) and (F) represent plant chemical traits. Gray boxplots represent control (non-mycorrhized plants), whereas blue boxplots represent plants that were inoculated with a mixture of arbuscular mycorrhizal fungi (AMF). Boxplots include the outliers (black dots), first quartile (black line), median (bold black line), and third quartile (black line), of data distribution.
4) *AMF effect on multivariate plant growth and chemical space –* Climatic and soil differences in the *Plantago* species’ niches were not found to correlate with neither the growth traits nor with the IGs matrix (Table S3). In contrast, we found that closely related species, regardless of the mycorrhizal status or ecological niche preferences, have IGs profiles that are more similar than distantly related species (Table S3), and we found no phylogenetic signal for the growth-related traits. Together, these patterns suggest a strong phylogenetic signal on the chemical profile regardless of inoculation/colonization with AMF. Through PERMANOVAs we found a species and a species by AMF interaction effect on the plant growth traits matrix (Figure S4A, Table S4), and species effect on the IGs matrix (Figure S4B, Table S4).
5) *Plant growth and chemical defense responsiveness to AMF colonization –* No significant correlation was found between plant growth-related phenotypic changes induced by AMF and AMF colonization level (Figure 3A, PGLS: r = 0.001, t = −0.004, p = 0.99, and phylogenetic signal for responsiveness of growth trait: λ = 0.25, p_λ_ = 0.33). However, we found that when species are highly mycorrhized, the species produced a more similar IG chemical profile structure than for species with low levels of mycorrhization (Figure 3B, PGLS: r = 0.52, t = −3.5, p = 0.007, and phylogenetic signal for responsiveness of chemical trait λ = 0.92, p_λ_ = 0.001). Finally, we found a negative relationship between growth and IG responsiveness to AMF colonization (Figure 3C, PGLS: r = 0.46, t = −2.5, p = 0.022), showing that those species which are more different in their IGs profiles when mycorrhized are those that are more similar in their growth-related phenotypes

1) *Variation in AMF colonization levels across species: phylogenetic and environmental drivers – Non-mycorrhizal control plants were observed free from mycorrhizal colonization, while total AMF root colonization, arbuscules and vesicles varied significantly across species (*Figure S5, *lm species effect for total colonization F_23,111_ = 3.57, p < 0.001; arbuscules; F_23,111_ = 2.19, p = 0.003 and vesicles F_23,111_ = 5.76, p < 0.001). We found a negative correlation between phylogenetic branch length and arbuscules (*Figure 1*C, r = -0.68, t_22_ = -4.5, p < 0.001, but not phylogenetic signal for arbuscules l = 0.41, p_l_ = 0.13). Moreover, while we observed a similar negative relationship, we found no significant effect for total colonization (*Figure S6*A, r = 0.17, t_22_ = -1.30, p = 0.21, no phylogenetic signal l = 0.24, p_l_ = 0.31), and vesicles (*Figure S6*B, r = -0.19, t_22_ = -0.44, p =0.66, no phylogenetic signal l = 0.29, p_l_ = 0.49). Finally, we found no correlation between pedo-climatic variables (coinertia axis 1, phylogenetic signal pedo-climatic niche l = 0.31, p_l_ = 0.28) with arbuscules (*Figure 1*D; PGLS: r = 0.18, t_22_ = 1.33, p = 0.20), with total colonization (*Figure S6*C; PGLS: r = 0.22, t_22_ = 0.05, p = 0.96) and with vesicles respectively (*Figure S6*D; PGLS: r = 0.17, t_22_ = -1.26, p = 0.22).*
2) *Phosphorus (P) and carbon to nitrogen (CN) amounts in leaves across species* – We found that high mycorrhizal species, on average when mycorrhized, had a 30% increase in P content in their leaves, whereas low mycorrhizal species has a 10% decrease in P content (Figure 1E; average effect size for high; 0.45 [2.39 to −1.47], and for low −0.47 [1.39 to −2.34] mycorrhizal species; PGLS: t = −2.62, p = 0.030). On the other hand, we found no effect of AMF colonization intensity for CN measured in leaves (Figure 1F; average effect size for high; −0.90 [1.23 to −3.02], and for low −0.17 [1.74 to −2.07] mycorrhizal species; PGLS: t = 0.79, p = 0.45).
3) *AMF effect on growth traits andiridoid glycosides (IGs)production –* We found that AMF changed the overall growth of plants by reducing total plant biomass by 10.7% (see negative effect in Table 1, Figure 2A), and by reducing plant biomass allocation to roots compared to shoots by 7.8%, compared to non-mycorrhizal control plants (Figure 2B and Table 1). However, we found no effect of AMF treatment on the leaf dry matter content (LDMC) and specific leaf area (SLA) (Figures 2C and 2D, Table 1). A total of 27 IGs were found across all species and treatments (Table S2), and the MCMCglmm models indicated that AMF had no overall effect on the total abundance, and richness of IGs across all species (Table 1).

**Table 1:** Effect of AMF treatment on plant growth traits and chemical defense concentration, number and diversity of IGs on 24 *Plantago* species as estimated with discriminant analysis using MCMCglmm with a Gaussian distribution

Dependent variable	Factor	Mean	L 95 ci	U 95 ci	ESS	pvalue
Total biomass	Intercept	3.00	−0.04	5.78	1000	0.046 ∗
	**AMF**	**0.46**	**0.23**	**0.69**	**1000**	**<0.001∗∗∗**
	Phylogeny (G)	11.35	5.55	18.17	1000	
	Residuals (R)	0.94	0.79	1.12	1000	
Root-shoot ratio	Intercept	0.39	−0.28	0.92	1000	0.21
	**AMF**	**0.16**	**0.11**	**0.21**	**1000**	**<0.001∗∗∗**
	G	0.52	0.23	0.86	1000	
	R	0.04	0.04	0.05	1000	
LDMC	Intercept	177.70	112.63	255.77	857.60	<0.001∗∗∗
	AMF	−0.26	−8.43	8.05	1000	0.92
	G	6519	2466	11495	1000	
	R	1163	949	1362	1000	
SLA	Intercept	17.77	9.88	26.23	1000	<0.001∗∗∗
	AMF	−0.09	−0.86	0.51	1000	0.81
	G	89.39	38.24	156.9	1000	
	R	7.758	6.455	9.215	894.8	
Total IGs	Intercept	4.05	2.60	5.38	1128	<0.001∗∗∗
	AMF	0.01	−0.15	0.14	1000	0.92
	G	2.56	1.09	4.43	1000	
	R	0.20	0.15	0.26	1102	
Number of IGs	Intercept	16.05	10.55	21.56	1000	<0.001∗∗∗
	AMF	−0.05	−0.72	0.54	1000	0.86
	G	42.75	20.37	72.67	896.1	
	R	4.62	3.50	5.90	1000	

Total iridoid glycosides (IGs) concentration was log+1 transformed. The G structure as the random effect of the species. Significant AMF effect based on posterior distributions and 95% credible intervals (CrI) are highlighted in bold. pvalues based on randomizations are also provided.

**Figure 3:**
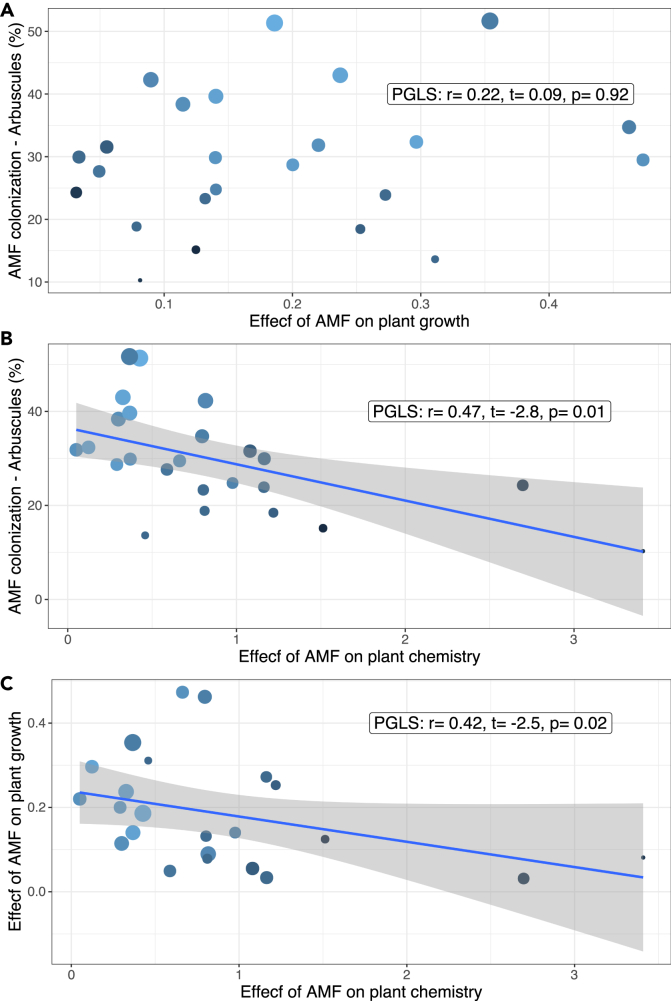
Species responses to AMF colonization (A and B) Shown are correlations between arbuscular mycorrhizal fungi (AMF) arbuscules colonization intensity and (A) the phenotypic distance (based on growth-related traits and as calculated from Figure S4A), and (B) the chemical distance (as calculated in Figure S4B) based on iridoid glycosides production for each *Plantago* species non-mycorrhized or with AMF. (C) shows the correlation between AMF-mediated growth changes and chemical changes across species. Dots are coded based on the root-to-tip distance of each species, with lighter colors indicating shorter branch length. Blue line and grey-shaded area around the line represent 95% confidence level interval for predictions from a linear model. Significance of the correlations is estimated using phylogenetic generalized least squares (PGLS) models.

## Discussion

We observed that across *Plantago* species, arbuscule colonization intensity decreases with increasing phylogenetic root-to-tip distance, but AMF colonization intensity was independent of the pedo-climatic niche of the species. Moreover, contrary to our prediction, species displaying the highest AMF colonization intensity changed their chemical profiles the least when colonized with AMF. Together, these effects resulted in a genus-wide trade-off between plant growth-related responses and the plant IGs production-responses to mycorrhizal colonization.

### Mycorrhizal colonization intensity declines over evolutionary time

Predicting across-species declines in mycorrhization might seem counterintuitive, if for instance we assume that all plant-mycorrhizae associations are beneficial for plants, and should therefore be favored by natural selection.^31^ However, not all plant-AMF associations are, or should be, beneficial. Several examples in the literature indeed indicate that AMF can be categorized along a parasitism to mutualism spectrum.^32^^,^^33^ Therefore, when the costs for plants of maintaining AMF in their roots become too high, natural selection during speciation events should favor individuals that form reduced symbiotic partnerships with AMF. Of interest, across the wider phylogeny of angiosperms, it was shown that multiple non-mycorrhizal families (e.g., Brassicaceae, Crassulaceae) generally derive from a mycorrhizal ancestor,^34^ whereas the reverse, the acquisition of mycorrhization from a non-mycorrhizal ancestor, has never been detected and is considered unlikely to happen.^35^ Moreover, the complete loss of mycorrhizae should first go through a weakening in colonization intensity.^35^ As we show, it appears that genetic relatedness, and the evolutionary trends therein, control the level of mycorrhization more than other factors, as we have shown here for pedo-climatic conditions. Particularly, we observed that species coming from similar climatic conditions, which may be more likely to grow in similar resource conditions, have disparate colonization intensities, thus suggesting a lack of ecological convergence within the *Plantago* genus for mycorrhization intensity. On the other hand, a study aiming at identifying predictors of global patterns of AMF colonization intensity emphasized the role of several climatic components and soil nutrient as drivers of root colonization intensity.^36^ By extracting coarse values (about 1 × 1 Km pixels) for soil and climatic variables, we indeed might have blurred finer levels of variation in microclimate and pedological forms, which would have masked within-species variation, ultimately accentuating the observed lack of among-species signal. Future studies should thus further address whether within species, levels of mycorrhization remain relatively constant (i.e., within species variation is smaller than across species variation), or whether the mycorrhization levels of different ecotypes is the result of a local adaptation to local climatic, and edaphic conditions, as well as the local fungal community.

Alternatively, different forces rather than climate adaptation, such as plants life history traits in relation to ecological succession, have also been proposed for studying variation in AMF colonization intensity and plant responsiveness to AMF.^15^ In our dataset, we have included six annual (or biennial) and 18 perennial species. Because there is indication that younger plants tend to sustain higher mycorrhizal colonization,^37^ we could expect perennials to be less mycorrhized, because they should reach maturity later, and they have more time to shed AMF structure from their roots. On the other hand, because perennials need to optimize their fitness over long periods of time, we could expect that the root-system-expanding properties of an AM network to be more helpful to perennial plants than to annuals. We indeed, found an indication that perennials have higher arbuscule colonization than annuals and that they are higher or not different for total or vesicle colonization (see Figure S7and Table S5). Other ecological factors, such as specific soil traits that were not available to us for this continent-wide analysis (e.g., CNP ratio, cation-exchange capacity),^38^ or plant species invasion potential,^39^ might also drive species-level variation in AMF colonization intensity. Declines in AMF intensity are thus common, and are related to phylogenetic history, however, the weak phylogenetic signal itself measured for AMF colonization intensity (λ = 0.42) suggests that other, not here measured, ecological factors, life-history traits, and plant-belowground traits are also driving across-species variation in how plants favor and maintain robust interactions with AMF communities.

Root traits themselves might also predict variation in mycorrhization intensity.^40^ For instance, plants that possess more fine root branching and shorter absorptive first-order roots harbor less AMF, whereas plants with thicker roots rely more on AMF to compensate for low absorptive surface. Accordingly, plants-AMF symbioses can be placed along a “collaborative gradient”.^41^ *Plantago* spp. might also follow such dynamics, because we have shown that higher level of arbuscules lead to higher phosphorus uptake, whereas lover levels of arbuscules characterize species with lower benefits in term of phosphorus uptake. Whether these effects, mediated by plant functional traits, also follow phylogenetic lines remains to be fully elucidated. A large-scale study showed that the best predictor of variation for the majority of root functional traits is the phylogenetic relatedness of the plants.^42^ This phylogenetic structuring of root functional traits may be present at a finer taxonomic across *Plantago* spp. and may partially explain the observed AM-arbuscules colonization de-escalation. However, to find the mechanistic reasons of why AMF colonization weakens in more derived species remains challenging. A future step in this direction might be to measure the selective pressure on the genes driving the symbiosis, such as STR2 and STR1.^43^

### Plant growth and chemical defense responsiveness to AMF

General theory suggests that the symbiosis with AMF should facilitate plant foraging capacities, and thus AMF should reduce allocation to root biomass and increase aboveground biomass, particularly when under nutrient limitations.^4^ In our controlled setting using potted plants, we indeed observed that plants reduced allocation to root biomass when mycorrhized (while maintaining similar aboveground biomass (AMF effect for aboveground biomass; 0.07 [-0.08 – 0.21], pMCMC = 0.31), which would indicate parsimonious use of resources (i.e., C allocation), when in presence of AMFs.^44^ That said, we also observed that plants were overall smaller with AMF. A large fraction of the gross primary production [up to 30%, ^45^] can be allocated to belowground symbiotic associations with AMF, and this fraction increases with decreasing nutrient availability,^46^ as plants then need to invest less in nutrient acquisition. Therefore, in our experiment, we might have observed a shift of carbon allocation from the shoots to the symbiotic fungi, because, although we maintained low levels of nutrients, soil fertility (about 10% organic matter) remained high enough for AMF to become carbon-sinks for our experimental plants.^47^

Concerning IGs, we observed no visible effect of AMFs on the full IG matrix across all *Plantago* species. These observations might contradict current trends in plant-microbe-insect interaction research which suggest that AMF colonization might increase plant resistance against insect pests^30^ by for instance increasing chemical defences.^48^ However, weak direct effects of AMF on plant chemical defenses have been previously shown.^16^ Other mechanisms of increased resistance might therefore come into play when plants are colonized by AMF. For instance, instead of producing costly metabolites continuously, mycorrhized plants can be favored by being primed to activate the defenses faster only when under attack by herbivores or pathogens.^49^ Because we only measured constitutive IGs on healthy plants, measuring the priming or higher inducibility effect was not feasible for this study, but undoubtedly feasible in future comparative phylogenetic studies using artificial elicitors (e.g., jasmonic acid or salicylic acid), or controlled herbivory manipulations.^50^ Nonetheless, we observed strong phylogenetic signal for IGs diversity across species, independently of whether plants were mycorrhized or not. This observation is in line with past findings, which demonstrated that IGs can be used as taxonomic markers for the different subgenera of the genus *Plantago*.^25^ In contrast, average species’ pedo-climatic niche did not correlate with IGs similarity, thus suggesting that phylogeny is a stronger driver of IGs diversity than the ecological niche of each species.

Furthermore with respect to IGs, we observed that in comparing AMF-colonized versus uncolonized plants, those taxa that had higher levels of AMF colonization showed less change in IG diversity. In other words, the degree of AMF colonization across species, which is to some extent phylogenetically controlled, also correlated with the degree to which a plant changes chemical defense profile when mycorrhized. More specifically, we would intuitively predict that higher AMF colonization, potentially providing more resources, would result in greater diversity of IGs, but our findings are in opposition to this prediction. This suggests two avenues for further research. First, the ability of plants to allow high degrees of AMF colonization is a pleiotropic trait that trades off with the ability to produce more diverse and more abundant IGs. This could be explained for example by a carbon allocation trade-off, because IGs are carbon-based molecules, and as we have discussed above, AMF can be carbon-sinks for many plant species.^51^^,^^52^ Whether this is true for other molecule classes, such as the nitrogen-containing alkaloids, needs to be confirmed in other systems. Second, and along other trade-off lines, we have here observed that AMF-induced changes in growth negatively correlate with changes in IGs. This might indicate that when mycorrhized, plant species are constrained to allocate resources to either functional traits related to growth, or to chemical defenses, but not to both simultaneously. Across-species growth-defense trade-offs have been widely postulated,^53^ which might also vary depending on the ecological context, such as the availability of resources,^54^ and thus, indirectly, the ability of maintaining beneficial symbioses with AMF.^55^ Therefore, whether within- or across species AMF-mediated growth-defense trade-offs are equally maintained needs further enquiry across a broader range of systems.

We show that AMF colonization intensity in *Plantago* species is tightly linked to the evolutionary history of the genus, and that more recently evolved species are on average less colonized than more ancestral species. The intensity of AMF colonization in turn dictates the degree of resources each species can allocate to growth or to chemical defenses. The history of land plants is intimately linked to the 400 M years old symbiosis with mycorrhizal fungi. It therefore appears that more recently evolved lineages tend to decrease their dependence to AMF, as shown here, as well as novel genera of families, as shown elsewhere, are relaxing the intensity of such associations. The causes of such declines in AMF-plant mutualism might be sought through resource-allocation trade-offs, physiological or evolutionary constraints, or through artificial selection experiments such as those naturally done during crop domestication.

### Limitations of the study

We here show that more recently evolved species are less mycorrhized. One limitation in relation to these findings, which we recommend including in future experiments, is to explore these effects by looking at arbuscular mycorrhizal symbiosis essential genes (e.g., STR2 and STR1) across multiple species to detect if these genes are under relaxed selective pressure in the low AMF colonized *Plantago* species. Secondly, we detected no effect of the macro-pedo-climatic niche of each species on AMF colonization intensity. However, finer-scale analyses might indicate a direct effect of soil properties on intraspecific variation for those *Plantago* species that have a wide distribution (e.g., *P. major*). Third, the correlation between AMF colonization and phylogenetic branch length was significant for arbuscular colonization but only a tendency was found for the correlation between phylogenetic branch length and total AMF root colonization. Further studies need to investigate the robustness of this relationship, especially because the occurrence of arbuscules is a more dynamic measure of AMF root colonization and depends on root age. Finally, future work should relate the observed findings, here obtained from a greenhouse experiment, with natural AMF colonization rates in field settings.

## STAR★Methods

### Key resources table

**Table:** 

REAGENT or RESOURCE	SOURCE	IDENTIFIER
**Chemicals, peptides, and recombinant proteins**		

KOH	Sigma-Aldrich	Cat#221473
Blue ink	Pelikan	Cat#301010
Lactic acid	Roth	Cat#8460.1
Glycerol	Roth	Cat#3783.2
Polyvinyl alcohol	Roth	Cat#1T2X.1
Nitric acid 65%	Merck	Cat#100456
Hydrogen peroxyde	Merck	Cat#3587191
Aucubin	Merck	Cat#479-98-1
Catalpol	Merck	Cat#50839
Methanol	Merck	Cat#200-659-6

**Deposited data**		

24 *Plantago* species ITS and trnLF sequences marker	GenBank	See: Iwanycki Ahlstrand et al.^56^
24 *Plantago* species occurrence records	Global Biodiversity Information facility	http://data.gbif.org
Climatic variables	Chelsa climatic data	https://chelsa-climate.org/
Soil physiochemical information	SoilGrids – ISRIC world Soil Information	https://soilgrids.org/
All raw and analyzed data deposited	This paper	https://datadryad.org/stash/share/UssC9sdnx-UXx3AfhaPdgr2Zd1RDOObvqS1vxafnhEo
See Table S2 for Iridoid Glycosides molecules identified	This paper	https://datadryad.org/stash/share/UssC9sdnx-UXx3AfhaPdgr2Zd1RDOObvqS1vxafnhEo

**Experimental models: Organisms/strains**		

The 24 *Plantago* species are provided in Table S1		
*Rhizophagus irregularis (syn. Rhizoglomus irregulare)*	SAF, Agroscope-CH	N/A
*Funneliformis mosseae*	SAF, Agroscope-CH	SAF # 11
*Claroideoglomus claroideum*	SAF, Agroscope-CH	N/A
*Divesispora celata*	SAF, Agroscope-CH	N/A

**Oligonucleotides**		

Primer for ITS and trnlf	Iwanycki Ahlstrand et al. 2019^56^	N/A

**Software and algorithms**		

MAFFT 7.2	https://mafft.cbrc.jp/alignment/software/	
Geneious	www.geneious.com	Version 9.1.8
MRBAYES	https://nbisweden.github.io/MrBayes/	Version 3.2.6
RAxML	https://cme.h-its.org/exelixis/web/software/raxml/	Version 8
Dictionary of Natural Products	CRC Press (USA)	Version 6.1.
ImageJ	https://ImageJ.nih.gov/ij/	N/A
R	R core Team	V. 4.1.1

**Other**		

Coupled plasma-optical emission spectroscopy 7300 DV ICP-OES	Perkin-Elmer	Cat#
Elemental analyser Flash2000	Thermo-Scientific	Cat#11230245
TissueLyser II	Qiagen	Cat#85300
UHPLC-QToF-MS	Waters	
Acquity BEH C18 column (50 × 2.1 mm, 1.7 μm particle)	Waters	Cat#186004661
Chlorophyll meter	Konica Minolta	ID: SPAD-502Plus

### Resource availability

#### Lead contact

Further information and requests for resources, data, materials, and methods should be directed to and will be fulfilled by the lead contact, Sergio Rasmann (sergio.rasmann@unine.ch).

#### Materials availability

The identified Iridoid Glycosides compounds are available in the supplemental information Table S2.

### Experimental model and subject details

#### Plant material: Plantago species

The genus *Plantago* is an optimal lineage of plants for investigating patterns of plant growth and defense responsiveness to AMF inoculation for multiple reasons. First, the genus *Plantago* has a worldwide distribution (see “method details” below) with several taxa occurring in temperate and Mediterranean biomes, as well as in the cold-desert regions.^57^ In the tropics, species are known to occur at high elevations, and several species occur as single island endemics^56^^,^^57^ (Figure 1B, Table S1). Second, all *Plantago* species tested to date are highly mycotrophic.^58^ Third, many species have been shown to produce IGs.^25^^,^^26^^,^^59^ For this study, we obtained seeds of 24 *Plantago* species representing each of the four major *Plantago* clades,^60^ including representatives from all continents (Table S1). As shown in Table S1, most of the seeds for the species tested originated from one unique source, therefore our within-species variation for all traits measured, including mycorrhization levels, was rather low. While this represents a major caveat for these type of macro-ecological studies, one major assumption that we make here is that within-species variation should be generally lower than across species variation, and therefore, which genotypes we chose should impact little the major findings across species, at the macroevolutionary scale [see e.g., ^61^^,^^62^^,^^63^. Moreover, although our sampling covers 12% of all species in the genus,^29^ it includes a broad spectrum of the phylogenetic (covering 10 out of 18 major *Plantago* sections, Figure S1), geographic and ecological diversity of the genus (Figures S2 and S3). Sequencing and phylogenetic reconstruction is described in the “method details” section.

#### Fungal organisms: Arbuscular mycorrhizal fungi inoculum

Two-weekold plants were inoculated with a mixture of four, broadly distributed and co-occurring AMF species: *R. irregularis*, *F. mosseae*, *C. claroideum* (order: Glomerales) and *D. celata* (order: Diversisporales). All species have been shown to have a global distribution,^64^^,^^65^ and all AMF species were obtained from the Swiss Collection of Arbuscular Mycorrhizal Fungi (SAF), Agroscope-CH (see Wagg et al.^66^ for details about origin and propagation of the inoculum). The inoculum consisted of a mixture of dry sandy substrate containing extra-radical spores and AMF-colonized root fragments. Un-inoculated, control plants were treated with the same substrate mixture, but free of AMF.^66^ We acknowledge that the different *Plantago* species used in the study might have co-evolved with different species or strains of AMF.^67^ However, despite the variable outcome of the plant-AMF symbiosis depending on the identity of the host plant or the symbiont, and the host selectivity for the AMF partners that occurs under natural conditions,^68^ it has been previously shown that under greenhouse conditions, almost any AMF is able to colonize any mycorrhizal plant species to some extent.^69^ Hence, both for practical reasons, and because we are working at the level of the same host plant genus with a recent evolutionary history (last estimation for most ancient *Plantago* divergence is approximately 16.7 Ma^56^), we opted to standardize the AMF community using a common inoculum for every plant species.

#### Common garden experiment

We ran a greenhouse common garden experiment in semi-controlled condition to measure the effect of AMF colonization on plant growth and IGs production. All seeds were germinated in Petri dishes laminated with moist filter papers at room temperature and in dark conditions. After germination, seedlings were transplanted into 13 cm width × 10 cm height plastic pots in a mixture of low nutrient substrate autoclaved twice at 121 C° for 20 min, the two cycles separated by 48h rest, composed of two-thirds of quartz sand and one-third of homemade compost soil (pH = 7.64, bioavailable p = 4 mg/kg, organic matter = 35%, CN = 11.5, CEC – Ca – Mg – K – Na – Al (cmol^(+)^/kg) = 29.5–0.016 – 0.002–0.0005 – 0–0). Before transplantation, the soil of each pot was homogenized with either the AMF inoculum (5% of the volume of the pot = 65 mL of inoculum) or the same substrate without AMF inoculum (the control). Half of the plants (n = 5–7 plants per species) received the AMF-containing substrate, while the other half (n = 5–7 plants per species) served as controls (see Table S1 for the number of replicates per species). Plants were grown for two months (July-August 2016) in an automatic vent-opening greenhouse at the Botanical Garden of Neuchâtel, Switzerland, under natural temperature and light conditions, in a fully-randomized scheme. Plants were watered every three days.

### Method details

#### Climatic and soil niche reconstruction

Occurrence records with geographical coordinate data for all species in our study were extracted from the Global Biodiversity Information Foundation (GBIF; http://data.gbif.org). Erroneous records were removed from the dataset. Ten of the 19 Chelsa climatic measures^70^ (BIO1 = Annual Mean Temperature, BIO3 = Isothermality (BIO2/BIO7) (∗ 100), BIO4 = Temperature Seasonality (standard deviation ∗100), BIO5 = Max Temperature of Warmest Month, BIO6 = Min Temperature of Coldest Month, BIO7 = Temperature Annual Range, BIO12 = Annual Precipitation, BIO13 = Precipitation of Wettest Month, BIO14 = Precipitation of Driest Month, BIO15 = Precipitation Seasonality (Coefficient of Variation); see PCA in Figure S1) were extracted for each species using the raster package^71^ in R (version 3.6.1).^72^ To avoid covariation of predictors and reduce the dimensionality of the climatic niche of the species we further condensed all the climatic variables using a Principal Component Analysis (PCA). The first two axis of the PCA explained together 83% of the variation. According to Horn’s Parallel Analysis performed in the paran package,^73^ the first axis was strongly correlated with temperature and precipitation (50% of variation explained), and therefore was selected for downstream analyses (Figure S2). Using the same geographical occurrence data used for climatic data extraction, soil physiochemical information was extracted from the Global Gridded Soil Information Database (SoilGrids – ISRIC world Soil Information.^74^ The median value of the 19 available variables at the depth interval ranging from 5 to 15 cm depth, the predominant bioactive zone for roots, were extracted. To avoid covariation of predictors and reduce the dimensionality of the soil niche of the species, we further condensed all the soil variables using a PCA. Five soil variables (absolute depth to bedrock, coarse fragment, SOC stock, soil H_2_O capacity pF 23, soil H_2_O capacity pF 25) were highly collinear and were removed from analysis while 14 soil variables (bulk density, clay content, depth to bedrock, H_2_O capacity, probability of occurrence of R horizon, SOC content, SOC density, sand content, saturated H_2_O content, silt content, soil H_2_O capacity pF 20, soil pH H_2_O and soil pH KC) were retained. The first two axes of the PCA explained 80% of the variation, and axes strongly correlated with organic carbon and pH (first axis 61% of variation explained, moving from organic and acid soils to non-organic basic soils along axis 1) and soil texture and CEC (second axis 19% of variation, from high nutrient clay soils to low nutrient sandy soils along axis 2) (Figure S3). Both axes were retained and used as a proxy of the soil niche of each species, based on Horn’s Parallel Analysis. To measure potential phylogenetic collinearity across pedo-climatic variable, we performed partial mantel tests (multi.mantel function in the phytools package^75^). We found no significant correlation between the climatic niche, soil niche and the species phylogenetic distance, indicating that closely-related species do not tend to occur in similar climatic regions (Partial Mantel Test, controlling for substrate; r = 0.06, p = 0.1) or similar soil substrate (Partial Mantel Test, controlling for climate; r = −0.02, p = 0.6).

#### Sequencing and phylogenetic reconstruction

For phylogenetic reconstruction, the ITS and trnlF introns were chosen for this study based on these two regions showing enough resolution across the major sub-genera in the genus *Plantago*.^56^^,^^60^ DNA sequences for seventy-four species, including the species *Litorella uniflora* L. were included in our full phylogenetic analyses (Data S1; Figure S1). Seventeen sequences were newly generated for this study (and uploaded to GenBank), and 85 and 45 sequences, respectively, previously generated in Rønsted et al.^60^ and Iwanycki Ahlstrand et al.^56^ were downloaded from GenBank. Genomic DNA was extracted from 15 to 20 mg of dried leaf tissue from herbarium vouchers made from plants grown under controlled conditions for this study (Data S1). Extraction, amplification and sequencing of ITS and trnlF regions were performed following Iwanycki Ahlstrand et al.^56^ Sequence alignment was performed using MAFFT 7.2^76^ using the software GENEIOUS 9.1.8. (www.geneious.com). The partitioned dataset comprising of sequences for 74 Plantago taxa was analyzed with MRBAYES 3.2.6.,^77^ as well as with RAxML^78^ following Iwanycki Ahlstrand et al.^56^ for both Bayesian and maximum likelihood analyses. Phylogenetic analyses were performed using 74 species to ensure that the 24 species selected for our study were resolved a well-supported topology similar to other recent studies, as well as to confirm species identities based on phylogenetic placement.^56^^,^^60^ The 50% majority rule consensus tree calculated in MRBAYES (Figure S1) was pruned to the 24 species used for analyses using function fix.poly in the RRphylo pacakage.^79^^,^^80^

#### AMF colonization intensity

Before oven-drying, soil particles were carefully cleared from roots and 1g of fresh young/secondary roots from each individual root system were randomly cut and stored at −20C° until the staining procedure was carried out to visualize AMF. Root staining consisted of 1) root clearing by KOH 10% during 10 minat 90 C° bath, 2) rinsing the KOH solution and acidifying with vinegar (5% acidity) at room temperature for 5 min, and 3) staining with a solution of 5% – blue ink/vinegar. The stained roots were submerged in glycerol in Eppendorf tubes and stored at 4 C° until slide preparation was undertaken. To estimate overall AMF colonization, ten root fragments of 1.5 cm length were placed vertically on a microscope slide. A solution of polyvinyl lacto-glycerol (PVLG), prepared by mixing 100 mL lactic acid, 100 mL ddH2O, 10 mL glycerol, and 16g polyvinyl alcohol powder at 80 Cº for 4 h, was added on the root fragments for microscopy visualization and preservation. AMF colonization intensity was estimated on n = 5 or 6 individual root system per Plantago species per AMF treatment (only fine roots were scored also non-mycorrhizal plant were scored for mycorrhizal colonization) following the intersection method.^80^

#### Phosphorours (P), carbon (C) and nitrogen (N) analyses

Between 3 and 4 replicates per AMF treatment and per species were selected. For total phosphorous (P), dry-leaf homogenates (<0.1g) were placed in Teflon vessels with de 5 mL nitric acid (65%, Suprapur, Merck KGaA, Germany) and 5 mL hydrogen peroxyde (33%), microwave digested using the following program: 600 W, 2 min; 0 W, 2 min; 450 W, 45 min, then analyzed by inductively coupled plasma-optical emission spectroscopy (7300 DV ICP-OES, Perkin-Elmer, USA). Carbon to nitrogen ratio (CN) was measured on the same leaf powder with an organic elemental analyser (Flash2000, Thermo Scientific, USA).

#### Iridoid glycosides (IGs) identification and quantification

For IGs analyses, one young yet fully-expanded leaf per plant was oven-dried at 40 °C for 48 h and ground to powder using an MM400 Retch TissueLyser (Qiagen, Hilden, Germany). Next, 10 mg of leaf powder per plant was extracted with 1.5 mL methanol, and the supernatant was diluted five times by adding 800 μL of MilliQ water to 200 μL of pure extract. IGs were separated using ultra-high performance liquid chromatography-time of flight mass spectrometry UHPLC-QToF-MS using an Acquity BEH C18 column from Waters (50 × 2.1 mm, 1.7 μm particle size) following the same protocol as in Bakhtiari et al.^81^ Absolute amounts of IGs were determined by external calibration using six standard solutions of catalpol (for catalpol quantification) and aucubin (for all other IGs quantification) at 0.2, 0.5, 2, 5, 10 and 20 μg mL-1. Concentrations were normalized to plant weight and expressed as μg mg-1 dry weight. IGs were identified based on their retention time and chemical formula by comparing them to previous chemical descriptions of Plantago species,^25^ or chemical database (Dictionary of Natural Products, CRC Press, USA, version 6.1. on DVD) (Table S2).

#### Plant functional trait measurement, root colonization

At the end of the two-months growing period, the following plant functional traits related to growth were measured according to Pérez-Harguindeguy et al.^36^; 1) root biomass (g dry weight (DW), 2) shoot biomass (g DW), 3) total plant biomass (g DW), 4) root-shoot ratio, 5) specific leaf area (SLA, mm^2^mg^−1^), 6) leaf dry matter content (LDMC, mg g^−1^), 7) chlorophyll content (SPAD). Specifically, SLA was calculated by dividing the area of the youngest fully-expanded leaf, estimated using ImageJ software (https://imagej.nih.gov/ij/) by its dry biomass. LDMC was calculated by dividing the dry biomass of the same leaf by its water-saturated fresh biomass. Chlorophyll content was measured on three youngest fully-expanded leaves per plant using an SPAD-502Plus chlorophyll meter (Konica Minolta, Investment Ltd., Tokyo, Japan). Finally, the whole plant was oven-dried at 40°C for 48h for measuring dry aboveground biomass, dry root biomass, and quantifying IGs in the leaves (see below). Next, to assess whether AMF colonization intensity could be related to variation in elemental composition (C, N, P) of Plantago leaves, we selected the five most (red arrows in Figure 1A) and the five least (green arrows in Figure 1B) colonized *Plantago* species across the phylogeny under investigation.

### Quantification and statistical analysis

#### IGs diversity and abundance indexes

We quantified the diversity (i.e., the number of individual componds) and abundance of all iridoid glycosides (IGs) found in all plants growing with and without AMF (see Table S2).

#### Statistical analyses

All statistical analyses were conducted using *R* (version 4.0.2).^72^

1) *Variation in AMF colonization levels across species: phylogenetic and environmental drivers* - First, we assessed the effect of *Plantago* species on colonization intensity using, after testing for model assumptions, one-way ANOVA. Next, we assessed the correlation between both phylogenetic history and environmental niche on AMF colonization levels. To test the effect of phylogenetic history, we used the root-to-tip distance (i.e., patristic distance calculated as the number of substitutions per site extracted with the *distRoot* function in the *adephylo* package.^82^ We regressed root-to-tip distance against AMF colonization levels using a linear model (*lm*). The phylogenetic signal was also calculated for the three AMF colonization variables using the function *phyloSignal* in the package *phylosignal*.^83^ For the environmental effect we performed a coinertia analysis between the retained climatic and pedological variables to check for a shared pedo-climatic structure using the *coin* function in the ‘*vegan*’ package.^84^ When significant, the coinertia analysis indicates a significant co-structuration (i.e., correlation) between the pedological and the climatic environments. Accordingly, we observed a significant positive correlation between the soil and climate matrices (r = 0.68, p = 0.001 based on 999 permutations), indicating, particularly along the first axis of the coinertia analysis, that *Plantago* species growing in colder and drier environments are also found to grow in deep soils with high pH, high bulk density, while species growing in warmer and more humid environments are also found to growing fertile soils. We regressed the coinertia axis 1 against AMF colonization levels using phylogenetic generalized least squares (PGLS) models, with the *λ* values estimated by maximum likelihood (ML) (*pGLS* function in the ‘*caper’* package^85^).
2) *P and carbon-to-nitrogen ratio (CN) –* To measure the effect of AMF colonization on P or CN in *Plantago* leaves, we first calculated effect sizes between non-mychorrized and mycorrhized plants, for each species individually, using Cohen’s d metric,^86^ as estimated with the ‘*effsize*’ package.^87^ In our case, a positive effect size indicates that the plants with AMF increase their P or N content compared to control, non-mycorrhized, plants. We next calculated group effect (high versus low mycorrhizal species) using phylogenetic generalized least squares (PGLS) models, with the *λ* values estimated by maximum likelihood (ML) (*pGLS* function in ‘*caper’* package^85^) on the pruned phylogenetic tree.
3) *AMF effect on univariate plant growth and chemical traits -* We tested the overall effect of AMF inoculation on growth traits (total biomass, root-shoot ratio, SLA, and LDMC), and chemical defense traits (total IG concentration, and the number of individual IGs) using a Monte Carlo Markov Chain generalized linear mixed model implemented in the ‘*MCMCglmm’* package (*MCMCglmm* with Gaussian distribution, and 10000 iterations).^88^ This Bayesian approach allows accounting for phylogenetic non-independence between species by including the phylogenetic variance-covariance matrix, built from a previously-converted to ultrametric tree with the function *force.ultrametric* in the package *‘phytools’*,^75^ as a random effect in the model, IGs, and growth traits as response variables. The phylogenetic signal was also estimated for all traits using the function *phyloSignal* in the package *phylosignal*.^83^
4) *AMF effect on multivariate plant growth and chemical space* –To estimate the phylogenetic, climate, and soil effects on growth traits and IGs, we performed multi-mantel tests with the *multi.mantel* function in the ‘*phytools’* package.^75^ For this we calculated species-level pairwise distances matrices of the phylogenetic, the climatic, the soil, the growth-related traits, and the IGs matrices using the *vegdist* function in the ‘*vegan’* package. Distance matrices for climate, soil and growth traits were calculated using Euclidean metrics, while IGs distances were calculated using Bray-Curtis metrics, since IGs were zero-inflated. The phylogenetic distance was calculated on the ultrametric tree using the *cophenetic* function. The *multi.mantel* test allows testing for the effect of phylogeny on traits while controlling for the effect of climate and soil and, vice versa, for testing the effect of climate while controlling for phylogenetic relatedness and soil, and so on. Analyses were done for both AMF-inoculated and control plants separately. Despite Mantel tests, as tools to investigate phylogenetic signals, have been shown to have poor statistical power, Mantel tests still remain the most favourable approach to measure correlations between phylogenetic distances and the whole growth or chemical profile of plants.^89^ Next, we tested for the effect of species by AMF treatment on both the multivariate plant growth trait matrix (Euclidean distance) and the chemical trait matrix (Bray-Curtis distance) using permutational multivariate ANOVA (PERMANOVA, using the *adonis* function in the ‘*vegan’* package).
5) *Plant growth and chemical defense responsiveness to AMF colonization –* To address how AMF influenced plant growth or chemical phenotypes across *Plantago* species, we projected the phenotypic distances for non-mycorrhized and for mycorrhized plants, for both plant growth traits and IGs as calculated above, on a 2-dimentional plane using non-metric multidimensional scaling (NMDS) in the package ‘*vegan’* (Figure S4). From this, we calculated the distance for each species between the control and the AMF treatment using the Euclidean metric formula. We regressed the calculated distances for plant growth traits and chemical traits separately, and for AMF colonization intensities across species using phylogenetic generalized least squares (PGLS) models, with the *λ* values estimated by maximum likelihood (ML) (*pGLS* function in ‘*caper’* package^85^). Finally, we performed PGLS regression analysis between AMF-mediated plant growth traits responsiveness and IG responsiveness to address potential trade-offs in responsive across plant growth or defense strategies.

## Data Availability

• Plant trait measurements, sequence data and AMF colonization intensity data have been deposited and are available in Dryad (https://datadryad.org/stash/share/UssC9sdnx-UXx3AfhaPdgr2Zd1RDOObvqS1vxafnhEo) and are listed in the key resources table. • This original research article does not contain any original code. • Any additional information required to reanalyse the data reported in this paper is available from the lead contact upon request. • The data associated with this publication is found at: https://datadryad.org/stash/share/UssC9sdnx-UXx3AfhaPdgr2Zd1RDOObvqS1vxafnhEo.

## Supplemental information

Document S1. Figures S1–S7 and Tables S1–S4

Data S1. Full Plantago list information, related to STAR Methods

## References

[1] Rillig M.C. (2004). Arbuscular mycorrhizae and terrestrial ecosystem processes. Ecol. Lett..

[2] Maherali H., Klironomos J.N. (2007). Influence of phylogeny on fungal community assembly and ecosystem functioning. Science.

[3] van der Heijden M.G.A., Klironomos J.N., Ursic M., Moutoglis P., Streitwolf-Engel R., Boller T., Wiemken A., Sanders I.R. (1998). Mycorrhizal fungal diversity determines plant biodiversity, ecosystem variability and productivity. Nature.

[4] Smith S.E., Read D.R. (2008).

[5] Corradi N., Bonfante P. (2012). The arbuscular mycorrhizal symbiosis: origin and evolution of a beneficial plant infection. PLoS Path.

[6] Pozo M.J., Azcón-Aguilar C. (2007). Unraveling mycorrhiza-induced resistance. Curr. Opin. Plant Biol..

[7] Mishra J., Singh R., Arora N.K. (2017). Alleviation of heavy metal stress in plants and remediation of soil by rhizosphere microorganisms. Front. Microbiol..

[8] Bunn R., Lekberg Y., Zabinski C. (2009). Arbuscular mycorrhizal fungi ameliorate temperature stress in thermophilic plants. Ecology.

[9] Johnson N.C., Graham J.H., Smith F.A. (1997). Functioning of mycorrhizal associations along the mutualism-parasitism continuum. New Phytol..

[10] Hoeksema J.D., Bever J.D., Chakraborty S., Chaudhary V.B., Gardes M., Gehring C.A., Hart M.M., Housworth E.A., Kaonongbua W., Klironomos J.N. (2018). Evolutionary history of plant hosts and fungal symbionts predicts the strength of mycorrhizal mutualism. Commun. Biol..

[11] Klironomos J.N. (2003). Variation in plant response to native and exotic arbuscular mycorrhizal fungi. Ecology.

[12] Reinhart K.O., Wilson G.W.T., Rinella M.J. (2012). Predicting plant responses to mycorrhizae: integrating evolutionary history and plant traits. Ecol. Lett..

[13] Gehring C.A., Mueller R.C., Haskins K.E., Rubow T.K., Whitham T.G. (2014). Convergence in mycorrhizal fungal communities due to drought, plant competition, parasitism and susceptibility to herbivory: consequences for fungi and host plants. Front. Microbiol..

[14] Peay K.G. (2016). The mutualistic niche: mycorrhizal symbiosis and community dynamics. Annu. Rev. Ecol. Evol. Syst..

[15] Koziol L., Bever J.D. (2015). Mycorrhizal response trades off with plant growth rate and increases with plant successional status. Ecology.

[16] Vannette R.L., Rasmann S. (2012). Arbuscular mycorrhizal fungi mediate below-ground plant–herbivore interactions: a phylogenetic study. Funct. Ecol..

[17] Bennett A.E., Bever J.D., Deane Bowers M. (2009). Arbuscular mycorrhizal fungal species suppress inducible plant responses and alter defensive strategies following herbivory. Oecologia.

[18] Iason G.R., Dicke M., Hartley S.E. (2012). The ecology of plant secondary metabolites: from genes to global processes.

[19] Agrawal A.A., Fishbein M. (2006). Plant defense syndromes. Ecology.

[20] De Deyn G.B., Biere A., van der Putten W.H., Wagenaar R., Klironomos J.N. (2009). Chemical defense, mycorrhizal colonization and growth responses in *Plantago lanceolata* L. Oecologia.

[21] Fontana A., Reichelt M., Hempel S., Gershenzon J., Unsicker S.B. (2009). The effects of arbuscular mycorrhizal fungi on direct and indirect defense metabolites of *Plantago lanceolata*. J. Chem. Ecol..

[22] Goverde M., van der Heijden M., Wiemken A., Sanders I., Erhardt A. (2000). Arbuscular mycorrhizal fungi influence life history traits of a lepidopteran herbivore. Oecologia.

[23] Kempel A., Schädler M., Chrobock T., Fischer M., van Kleunen M. (2011). Tradeoffs associated with constitutive and induced plant resistance against herbivory. Proc. Natl. Acad. Sci. USA.

[24] Rasmann S., Bennett A., Biere A., Karley A., Guerrieri E. (2017). Root symbionts: powerful drivers of plant above- and belowground indirect defenses. Insect Sci..

[25] Rønsted N., Franzyk H., Mølgaard P., Jaroszewski J.W., Jensen S.R. (2003). Chemotaxonomy and evolution of *plantago*. Plant Syst. Evol..

[26] Bowers M.D., Stamp N.E. (1993). Effects of plant age, genotype and herbivory on plantago performance and chemistry. Ecology.

[27] Deane Bowers M., Collinge S.K., Gamble S.E., Schmitt J. (1992). Effects of genotype, habitat, and seasonal variation on iridoid glycoside content of Plantago lanceolata (Plantaginaceae) and the implications for insect herbivores. Oecologia.

[28] Marak H.B., Biere A., Van Damme J.M. (2002). Two herbivore-deterrent iridoid glycosides reduce the in-vitro growth of a specialist but not of a generalist pathogenic fungus of Plantago lanceolata L. Chemoecology.

[29] Rahn K. (1996). A phylogenetic study of the Plantaginaceae. Bot. J. Linn. Soc..

[30] Gruden K., Lidoy J., Petek M., Podpečan V., Flors V., Papadopoulou K.K., Pappas M.L., Martinez-Medina A., Bejarano E., Biere A. (2020). Ménage à trois: unraveling the mechanisms regulating plant–microbe–arthropod interactions. Trends Plant Sci..

[31] Brundrett M. (2004). Diversity and classification of mycorrhizal associations. Biological reviews.

[32] Karst J., Marczak L., Jones M.D., Turkington R. (2008). The mutualism–parasitism continuum in ectomycorrhizas: a quantitative assessment using meta-analysis. Ecology.

[33] Johnson N.C., Graham J.H. (2013). The continuum concept remains a useful framework for studying mycorrhizal functioning. Plant Soil.

[34] Feijen F.A.A., Vos R.A., Nuytinck J., Merckx V.S.F.T. (2018). Evolutionary dynamics of mycorrhizal symbiosis in land plant diversification. Sci. Rep..

[35] Maherali H., Oberle B., Stevens P.F., Cornwell W.K., McGlinn D.J. (2016). Mutualism persistence and abandonment during the evolution of the mycorrhizal symbiosis. Am. Nat..

[36] Soudzilovskaia N.A., Douma J.C., Akhmetzhanova A.A., van Bodegom P.M., Cornwell W.K., Moens E.J., Treseder K.K., Tibbett M., Wang Y.P., Cornelissen J.H.C. (2015). Global patterns of plant root colonization intensity by mycorrhizal fungi explained by climate and soil chemistry. Glob. Ecol. Biogeogr..

[37] Werner G.D.A., Kiers E.T. (2015). Order of arrival structures arbuscular mycorrhizal colonization of plants. New Phytol..

[38] Oehl F., Laczko E., Oberholzer H.-R., Jansa J., Egli S. (2017). Diversity and biogeography of arbuscular mycorrhizal fungi in agricultural soils. Biol. Fertil. Soils.

[39] Reinhart K.O., Lekberg Y., Klironomos J., Maherali H. (2017). Does responsiveness to arbuscular mycorrhizal fungi depend on plant invasive status?. Ecol. Evol..

[40] Wen Z., Li H., Shen Q., Tang X., Xiong C., Li H., Pang J., Ryan M.H., Lambers H., Shen J. (2019). Tradeoffs among root morphology, exudation and mycorrhizal symbioses for phosphorus-acquisition strategies of 16 crop species. New Phytol..

[41] Weigelt A., Mommer L., Andraczek K., Iversen C.M., Bergmann J., Bruelheide H., Fan Y., Freschet G.T., Guerrero-Ramírez N.R., Kattge J. (2021). An integrated framework of plant form and function: the belowground perspective. New Phytol..

[42] Valverde-Barrantes O.J., Freschet G.T., Roumet C., Blackwood C.B. (2017). A worldview of root traits: the influence of ancestry, growth form, climate and mycorrhizal association on the functional trait variation of fine-root tissues in seed plants. New Phytol..

[43] Radhakrishnan G.V., Keller J., Rich M.K., Vernié T., Mbadinga Mbadinga D.L., Vigneron N., Cottret L., Clemente H.S., Libourel C., Cheema J. (2020). An ancestral signalling pathway is conserved in intracellular symbioses-forming plant lineages. Nat. Plants.

[44] Veresoglou S.D., Menexes G., Rillig M.C. (2012). Do arbuscular mycorrhizal fungi affect the allometric partition of host plant biomass to shoots and roots? A meta-analysis of studies from 1990 to 2010. Mycorrhiza.

[45] Hobbie E.A. (2006). Carbon allocation to ectomycorrhizal fungi correlates with belowground allocation in culture studies. Ecology.

[46] Lilleskov E.A., Fahey T.J., Horton T.R., Lovett G.M. (2002). Belowground ectomycorrhizal fungal community change over a nitrogen deposition gradient in Alaska. Ecology.

[47] Verlinden M.S., Ven A., Verbruggen E., Janssens I.A., Wallander H., Vicca S. (2018). Favorable effect of mycorrhizae on biomass production efficiency exceeds their carbon cost in a fertilization experiment. Ecology.

[48] Jung S.C., Martinez-Medina A., Lopez-Raez J.A., Pozo M.J. (2012). Mycorrhiza-induced resistance and priming of plant defenses. J. Chem. Ecol..

[49] Prime-A-Plant Group, Conrath U., Beckers G.J.M., García-Agustín P., García-Agustín P., Jakab G., Mauch F., Newman M.A., Pieterse C.M.J., Poinssot B. (2006). Priming: getting ready for battle. Mol. Plant Microbe Interact..

[50] Bakhtiari M., Glauser G., Rasmann S. (2018). Root JA induction modifies glucosinolate profiles and increases subsequent aboveground resistance to herbivore attack in *Cardamine hirsuta*. Front. Plant Sci..

[51] Bennett A.E., Bever J.D. (2009). Trade-offs between arbuscular mycorrhizal fungal competitive ability and host growth promotion in Plantago lanceolata. Oecologia.

[52] Mraja A., Unsicker S.B., Reichelt M., Gershenzon J., Roscher C. (2011). Plant community diversity influences allocation to direct chemical defence in Plantago lanceolata. PLoS One.

[53] Züst T., Agrawal A.A. (2017). Trade-offs between plant growth and defense against insect herbivory: an emerging mechanistic synthesis. Annu. Rev. Plant Biol..

[54] Coley P.D., Bryant J.P., Chapin F.S. (1985). Resource availability and plant antiherbivore defense. Science.

[55] Vannette R.L., Hunter M.D. (2011). Plant defence theory re-examined: nonlinear expectations based on the costs and benefits of resource mutualisms. J. Ecol..

[56] Iwanycki Ahlstrand N., Verstraete B., Hassemer G., Dunbar-Co S., Hoggard R., Meudt H.M., Rønsted N. (2019). Ancestral range reconstruction of remote oceanic island species of Plantago (Plantaginaceae) reveals differing scales and modes of dispersal. J. Biogeogr..

[57] Van der Aart P., Vulto J., Soekarjo R., van Damme J. (1992). Plantago: A Multidisciplinary Study.

[58] Hart M.M., Reader R.J. (2002). Taxonomic basis for variation in the colonization strategy of arbuscular mycorrhizal fungi. New Phytol..

[59] Iwanycki Ahlstrand N., Havskov Reghev N., Markussen B., Bruun Hansen H.C., Eiriksson F.F., Thorsteinsdóttir M., Rønsted N., Barnes C.J. (2018). Untargeted metabolic profiling reveals geography as the strongest predictor of metabolic phenotypes of a cosmopolitan weed. Ecol. Evol..

[60] Rønsted N., Chase M.W., Albach D.C., Bello M.A. (2002). Phylogenetic relationships within Plantago (Plantaginaceae): evidence from nuclear ribosomal ITS and plastid trnL-F sequence data. Bot. J. Linn. Soc..

[61] Agrawal A.A., Fishbein M., Halitschke R., Hastings A.P., Rabosky D.L., Rasmann S. (2009). Evidence for adaptive radiation from a phylogenetic study of plant defenses. Proc. Natl. Acad. Sci. USA.

[62] Rasmann S., Agrawal A.A. (2011). Latitudinal patterns in plant defense: evolution of cardenolides, their toxicity and induction following herbivory. Ecol. Lett..

[63] Pellissier L., Litsios G., Fishbein M., Salamin N., Agrawal A.A., Rasmann S. (2016). Different rates of defense evolution and niche preferences in clonal and nonclonal milkweeds (Asclepias spp.). New Phytol..

[64] Öpik M., Moora M., Liira J., Zobel M. (2006). Composition of root-colonizing arbuscular mycorrhizal fungal communities in different ecosystems around the globe. J. Ecol..

[65] Davison J., Moora M., Öpik M., Adholeya A., Ainsaar L., Bâ A., Burla S., Diedhiou A.G., Hiiesalu I., Jairus T. (2015). Global assessment of arbuscular mycorrhizal fungus diversity reveals very low endemism. Science.

[66] Wagg C., Jansa J., Schmid B., Van Der Heijden M.G.A. (2011). Belowground biodiversity effects of plant symbionts support aboveground productivity. Ecol. Lett..

[67] Johnson N.C., Wilson G.W.T., Bowker M.A., Wilson J.A., Miller R.M. (2010). Resource limitation is a driver of local adaptation in mycorrhizal symbioses. Proc. Natl. Acad. Sci. USA.

[68] Werner G.D.A., Kiers E.T. (2015). Partner selection in the mycorrhizal mutualism. New Phytol..

[69] Scheublin T.R., Ridgway K.P., Young J.P.W., van der Heijden M.G.A. (2004). Nonlegumes, legumes, and root nodules harbor different arbuscular mycorrhizal fungal communities. Appl. Environ. Microbiol..

[70] Karger D.N., Conrad O., Böhner J., Kawohl T., Kreft H., Soria-Auza R.W., Zimmermann N.E., Linder H.P., Kessler M. (2017). Climatologies at high resolution for the earth’s land surface areas. Sci. Data.

[71] Hijmans R.J. (2019).

[72] R Development Core Team (2020).

[73] Dinno A. (2009). Implementing horn's parallel analysis for principal component analysis and factor analysis. STATA J..

[74] Hengl T., Mendes De Jesus J., Heuvelink G.B.M., Ruiperez Gonzalez M., Kilibarda M., Blagotić A., Shangguan W., Wright M.N., Geng X., Bauer-Marschallinger B. (2017). SoilGrids250m: global gridded soil information based on machine learning. PLoS One.

[75] Revell L.J. (2012). phytools: an R package for phylogenetic comparative biology (and other things). Methods Ecol. Evol..

[76] Katoh K., Standley D.M. (2013). MAFFT multiple sequence alignment software version 7: improvements in performance and usability. Mol. Biol. Evol..

[77] Ronquist F., Huelsenbeck J.P. (2003). MrBayes 3: Bayesian phylogenetic inference under mixed models. Bioinformatics.

[78] Stamatakis A. (2014). RAxML version 8: a tool for phylogenetic analysis and post-analysis of large phylogenies. Bioinformatics.

[79] Castiglione S., Tesone G., Piccolo M., Melchionna M., Mondanaro A., Serio C., Di Febbraro M., Raia P. (2018). A new method for testing evolutionary rate variation and shifts in phenotypic evolution. Methods Ecol. Evol..

[80] Giovannetti M., Mosse B. (1980). An evaluation of techniques for measuring vesicular arbuscular mycorrhizal infection in roots. New Phytol..

[81] Bakhtiari M., Formenti L., Caggìa V., Glauser G., Rasmann S. (2019). Variable effects on growth and defense traits for plant ecotypic differentiation and phenotypic plasticity along elevation gradients. Ecol. Evol..

[82] Jombart T., Balloux F., Dray S. (2010). adephylo: new tools for investigating the phylogenetic signal in biological traits. Bioinformatics.

[83] Keck F., Rimet F., Bouchez A., Franc A. (2016). phylosignal: an R package to measure, test, and explore the phylogenetic signal. Ecol. Evol..

[84] Oksanen J., Blanchet F.G., Kindt R., Legendre P., Minchin P.R., O'Hara R.B., Simpson G.L., Solymos P., Stevens M.H.H., Wagner H. (2013). http://vegan.r-forge.r-project.org/.

[85] Orme D., Freckleton R., Thomas G., Petzoldt T., Fritz S., Isaac N. (2013).

[86] Cohen J.A. (1988).

[87] Torchiano M. (2018).

[88] Hadfield J.D. (2010). MCMC methods for multi-response generalized linear mixed models: the MCMCglmm R Package. J. Stat. Softw..

[89] Harmon L.J., Glor R.E. (2010). Poor statistical performance of the mantel test in phylogenetic comparative analyses. Evolution.

