# Supplementary material for: Macroevolutionary decline in mycorrhizal colonization and chemical defense responsiveness to mycorrhization
Source: iScience. 2023 Apr 10;26(5):106632. doi: 10.1016/j.isci.2023.106632 (PMC10165190; doi:10.1016/j.isci.2023.106632)
Supplement: Document S1. Figures S1–S7 and Tables S1–S4 [file mmc1.pdf]

iScience, Volume *26*

Supplemental information

Macroevolutionary decline in mycorrhizal colonization and chemical defense responsiveness to mycorrhization

Ludovico Formenti, Natalie Iwanycki Ahlstrand, Gustavo Hassemer, Gaëtan Glauser, Johan van den Hoogen, Nina Rønsted, Marcel van der Heijden, Thomas W. Crowther, and Sergio Rasmann

Table S1. Information about the plant material included in the study, **related to STAR methods and Figure 1**. Botanical gardens that provided plant seeds: OBS = Orto Botanico Siena (IT), MNHN = Museum National d'Histoire Naturelle de Paris (FR), BGTAU = Botanic Garden of Tel Aviv University (IL), JBG = Jerusalem Botanical Garden (IL), NBGV = National Botanical Garden Vacratot (HU), CJBN = Conservatoire et Jardins Botaniques de Nancy (FR), HBUL = Hortus Botanicus Universitatis Lasi (RO), BGK = Botanical Garden of Komaroba (RU), UFA = commercial seeds provided by UFA (CH), BGSG = Botanical Garden of St-Gallen (CH). N samples column includes the number of samples for plants trait and chemistry for C/AMF – colonization.

Species	Provider	Wild Provenance	Voucher (ID)	Distribution	N samples	Life form
P. afra **L.**	OBS	Unknown	XX-0-SIENA-01670	Mediterranean	6/5 - 4	annual
P. albicans **L.**	MNHN	Leucrate - FR	MNHN-JB-71052	Mediterranean	6/5 - 5	perennial
P. alpina **L.**	MNHN	Val d'Aosta - IT	IT-0-NCY-19818101W	Europe	6/6 - 6	perennial
P. altissima **L.**	NBGV	Unknown	1159	Europe, N Africa	5/5 - 5	perennial
P. arborescens **Poir.**	JBG	Unknown	Unknow ID	Macaronesia	6/6 - 6	perennial
P. arenaria **Waldst. & Kit.**	OBS	Unknown	XX-0-SIENA-01671	Mediterranean	4/2 - 2	annual
P. asiatica **L.**	NBGV	Unknown	1160	S & E Asia	6/6 - 6	perennial
P. atrata **Hoppe.**	CJBN	Mont Touazey, Jura - FR	FR-0-NCY-19741999W	Europe, W Asia	5/5 - 5	perennial
P. australis **Lam.**	Unknown	Unknown	Unknow ID	America	6/6 - 6	perennial
P. bellardii **All.**	CJBN	Calvi - FR	MNHN-JB-9247	Mediterranean	6/6 - 6	annual
P. coronopus **L.**	MNHN	Leucrate - FR	MNHN-JB-9250	Medit., Eu.	5/6 - 4	annual
P. crassifolia **Forsskal**	BGTAU	Cult.e, Akko (Acre) Plain - IS	2014.0361	Medit., S Africa	6/6 - 6	perennial
P. cretica **L.**	BGTAU	Mount Carmel - IS	2012.0949	E Mediterranean	6/6 - 6	annual
P. lagopus **L.**	MNHN	Leucrate - FR	MNHN-JB-36010	Mediterranean	6/6 - 6	annual
P. lanceolata **L.**	UFA	CH	Unknow ID	Cosmopolite	6/6 - 6	perennial
P. macrorhiza **Poir.**	MNHN	Bizerte - TN	MNHN-JB-49585	Mediterranean	6/6 - 6	perennial
P. major **L.**	MNHN	Avoriaz - FR	MNHN-JB-65756	Cosmopolite	6/6 - 6	perennial
P. maritima **L.**	MNHN	Plounévez Lochrist - FR	MNHN-JB-9257	Cosmopolite	7/5 - 4	perennial
P. media **L.**	NBGV	Unknown	1166	Europe, C Asia	6/6 - 6	perennial
P. nivalis **Boiss.**	CJBN	Sierra Nevada - ES	XX-0-NCY-19740962G	S Spain	5/5 - 5	perennial
P. raoulii **Decne.**	BGSG	New Zeland	Unknow ID	New Zeland	6/5 - 6	perennial
P. schwarzenbergiana **Schur.**	HBUL	Valea Ilenei Leg. Ana Cojocariu - RO	RO-0-IAGB20141927W	E Europe, Balkans	6/6 - 6	perennial
P. sempervirens **Crantz.**	MNHN	Vebron - FR	MNHN-JB-9263	SW Europe	6/6 - 5	perennial
P. subulata **L.**	CJBN	Corse - FR	Fr-0-NCY-19760102W	Mediterranean	7/5 - 3	perennial

Table S2. Iridoid glycosides (IGs) detected across 24 species of *Plantago* used in the study, **related to STAR methods**. Putative = putative identification based on mass spectral profiles.

Identification level	Compound Name	Classification	Formula	Exact mass
**IGS detected in *Plantago* spp.**	10-O-Acetylgeniposidic acid	Precursor	C18H24O11	416.131865
	10-Acetoxymajoroside	Δ- 8,9	C19H26O12	446.14243
	10-Benzoylcatalpol	Normal	C22H26O11	466.147515
	10-Hydroxymajoroside	Δ- 8,9	C17H24O11	404.131865
	3,4-Dihydroaucubin	Normal	C15H24O9	348.34566
	Alpinoside	NA	C18H24O11	416.131865
	Arborescoside	Δ- 8,9	C17H24O10	388.13695
	Asperuloside	Others	C18H22O11	414.116215
	Aucubin	Normal	C15H22O9	346.126385
	Auroside	Precursor	C17H26O11	406.147515
	Bartsioside	Normal	C15H22O8	330.13147
	Caryoptoside	Precursor	C17H26O11	406.147515
	Catalpol	Normal	C15H22O10	362.1213
	Deacetylalpinoside	Precursor	C16H22O10	374.1213
	Deacetylasperuloside	Others	C16H20O10	372.10565
	Desacetylhookerioside	Δ- 8,9	C22H32O15	536.174125
	Epiloganic acid	Precursor	C16H24O10	376.13695
	Gardoside	Precursor	C16H22O10	374.1213
	Geniposidic acid	Precursor	C16H22O10	374.1213
	Glucosylaucubin	Normal or 5-OH	C21H32O14	508.17921
	Hookerioside	Δ- 8,9	C24H34O16	578.18469
	Majoroside	Δ- 8,9	C17H24O10	388.13695
	Melittoside	5-OH	C21H32O15	524.174125
	Monomelittoside	5-OH	C15H22O10	362.1213
	Mussaenosidic acid	Precursor	C16H24O10	376.13695
	Plantarenaloside	Others	C16H24O9	360.142035
	Plantarenaloside isomer	Others	C16H24O9	360.142035
	Strictoloside	5-OH	C16H22O12	406.11113
**Known identified IGS**	8-O-Acetylshanzhiside	Others	C18H26O12	434.14243
	Aucubigenin;10-O-[3-Methylbutanoyl], 1-O-β-D-glucopyranoside	Others	C20H30O10	430.1839
	Iridoidal gentiobioside	Others	C22H36O12	492.22068
	Methylscutelloside	Others	C16H26O11	394.147515
	Nemorososide	Others	C25H38O11	514.241415
	Scyphiphin D	precursor (dimer)	C32H42O19	730.232035
**Putative IGS**	IG 1 Isomer A	Precursor	C16H26O8	346.16277
	IG 1 Isomer B	Precursor	C16H26O8	346.16277
	IG 1 Isomer C	Precursor	C16H26O8	346.16277
	IG 2 Isomer A	Others	C16H28O8	348.17842
	IG 2 Isomer B	Others	C16H28O8	348.17842
	IG 3	5-OH	C15H24O11	380.131865
	IG 4 Isomer A	Others	C16H22O11	390.343
	IG 4 Isome B	Others	C16H22O11	390.343
	IG 5	Precursor	C17H26O10	390.1526
	IG 6 Isomer A	Others	C16H24O11	392.131865
	IG 6 Isomer B	Others	C16H24O11	392.131865
	IG 6 Isomer C	Others	C16H24O11	392.131865
	IG 7	NA	C19H28O11	432.163165
	IG 8	Othres	C18H28O12	436.15808
	IG 9	Others	C20H30O13	478.168645
	IG 10	Others	C25H28O12	520.15808
	IG 11	Others	C22H34O14	522.19486
	IG 12	NA	C26H32O13	552.184295

Table S3. Multiple Mantel result table, **related to results section 4)**. *For* testing the effect of phylogenetic distance, climatic distance and soil distance with growth and defence traits distance matrices across *Plantago* species. Multiple correlation analyses between matrices were done for AMF treated and untreated (Control) plants separately and AMF treatment. R^2 is the multiple-R square value showing the degree of correlation for the overall multiple correlation model, while p is the probability associated with R^2 based on 999 permutations for the overall multiple correlation model. $p < 0.05$ are highlighted in bold and $*p < 0.05$, $**p < 0.01$ and $***p < 0.001$. The Multiple mantel test (based on partial Mantel test) was performed to test the correlation of the phylogenetic relationship, climatic niche or soil niche with growth and chemical defense distance matrices while controlling the effect of the other matrices.

Traits distance matrix	AMF treatment	Distance matrices	Estimate	t value	Pr(>|t|)	R^2	p	F
**Growth**	Un-inoculated	Intercept (I)	52.43	8.36	0.29	0.01	0.91	0.67
		Phylogeny (P)	-12.57	-0.22	0.82			
		Climate (C)	0.00	0.51	0.76			
		Soil (S)	0.00	-1.58	0.29			
	Mycorrhizal	I	47.67	7.33	0.62	0.04	0.07	3.96
		P	109.66	1.88	0.05			
		C	0.00	-2.98	0.05			
		S	0.00	-0.16	0.92			
**Chemistry (IG)**	Un-inoculated	I	5.86	15.52	1.00	0.17	**0.004**	18.45
		P	1.63	6.29	**0.001**			
		C	--2.43	-3.15	0.140			
		S	6.57	2.71	0.197			
	Mycorrhizal	I	5.77	15.17	1.00	0.8	**0.004**	19.38
		P	1.77	6.78	**0.001**			
		C	-2.26	-2.90	0.18			
		S	5.50	2.25	0.27			

Bold font highlight significant effects ($p < 0.05$)

Table S4. PERMANOVA table, **related to results section 4).** For testing the interactive effects of *Plantago* plant species and AMF treatment (with or without AMF) on plant growth traits or iridoid glycosides (IGs).

**Traits**	**Factor**	**Df**	**SSQ**	**F**	**R2**	**Pr(>F)**
**Growth traits**	Species	23	2.73	19.35	0.64	**<0.001**
	AMF	1	0.00	0.34	0.00	0.73
	Species * AMF	23	0.21	1.45	0.05	**0.04**
	Residuals	218	1.34	0.31		
	Total	265	4.28			
**IGs**	Species	26	40.88	22.95	0.83	**<0.001**
	AMF	1	0.04	0.62	0.00	0.83
	Species * AMF	26	1.78	1.00	0.04	0.48
	Residuals	99	6.78	0.14		
	Total	152	49.48			

Bold font highlight significant effects ($p < 0.05$)

Table S5. Results table based on Markov chain Monte Carlo Sampler for Multivariate Generalised Linear Mixed Models (MCMCglmm), **related to the Discussion section**. Effect of life form (annual and biannual versus perennial) on total AMF mycorrhization, the colonization by arbuscules, and the amount of AMF vesicles in 24 *Plantago* species was estimated with discriminant analysis using MCMCglmm with a gaussian distribution. The G structure as the random effect of the species. Significant AMF effect based on posterior distributions and 95% credible intervals (CrI) are highlighted in bold. p-values based on randomizations are also provided.

Dependent variable	Factor	Mean	L 95 ci	U 95 ci	ESS	p-value
**Total mycorrhizal colonization (%)**	Intercept	61.214	38.125	79.059	1000	**0.002**
	**Life form**	8.262	-7.097	25.104	835.8	0.278
	Phylogeny (G)	339.3	38.01	815.5	506.5	
	Residuals (R)	410.5	307.2	535.5	799.7	
**Mycorrhizal colonization - arbuscules (%)**	Intercept	20.735	11.316	30.410	842.1	**0.002**
	**Life form**	**11.988**	**2.815**	**21.300**	**762.5**	**<0.05**
	G	38.93	1.76e-09	129.7	34.25	
	R	278.6	205.1	363.6	63.65	
**Mycorrhizal colonization - vescicles (%)**	Intercept	11.842	-2.597	25.195	1000	0.108
	**Life form**	5.805	-3.029	15.185	1000	0.176
	G	211.9	62.49	431.8	771.9	
	R	90.91	67.44	118.7	1000	

Bold font highlight significant effects ($p < 0.05$)

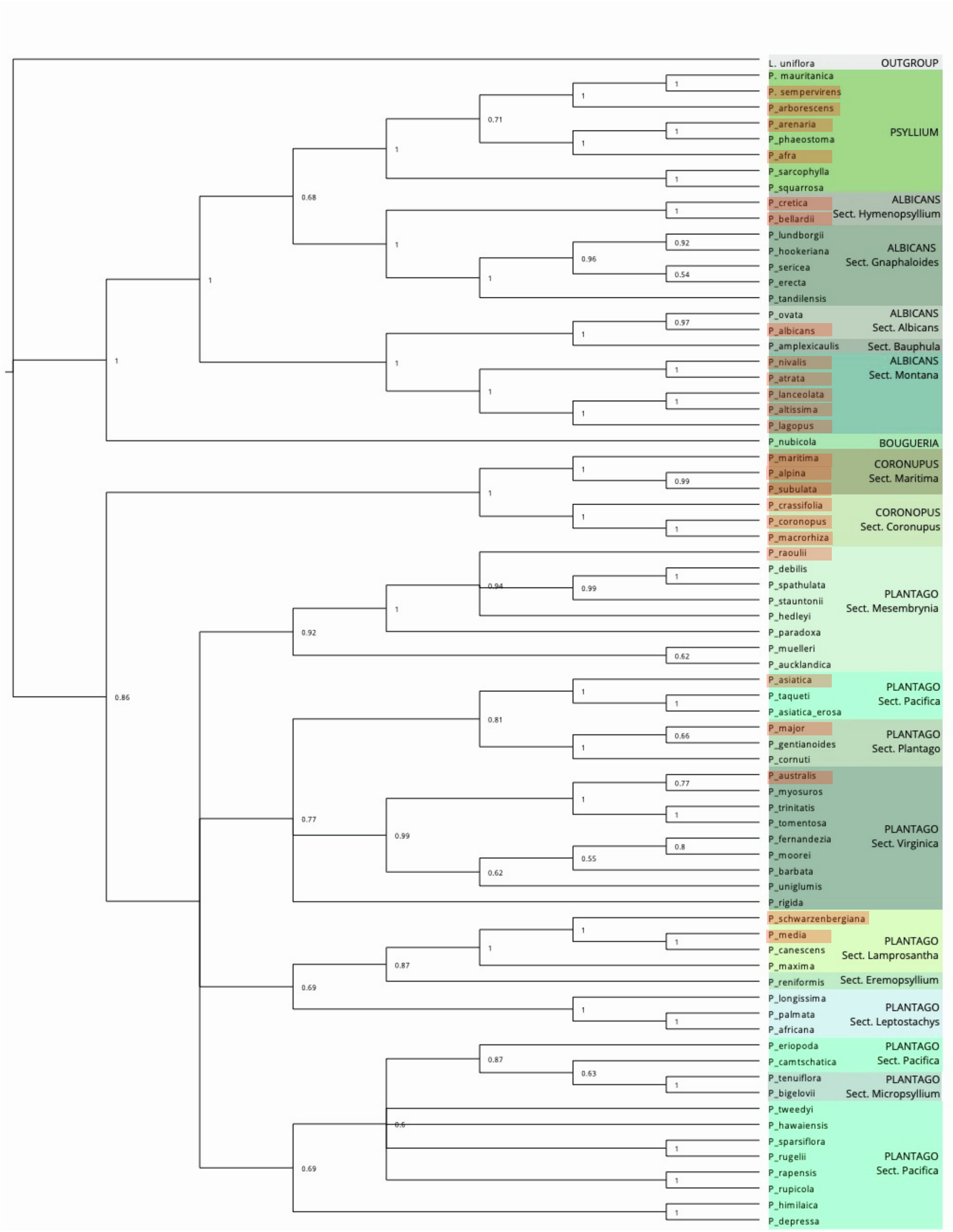

Figure S1. The 50% majority rule cladogram from the MRBAYES analyses for 74 *Plantago* species including out group (*Litorella uniflora),* **related to STAR methods**. Posterior probabilities are listed at the branch nodes. Taxonomic sections of the genus *Plantago* are shown as different colours. This phylogenetic resolution was pruned to the 24 species (highlighted in red) for the phylogenetic tree used in the study.

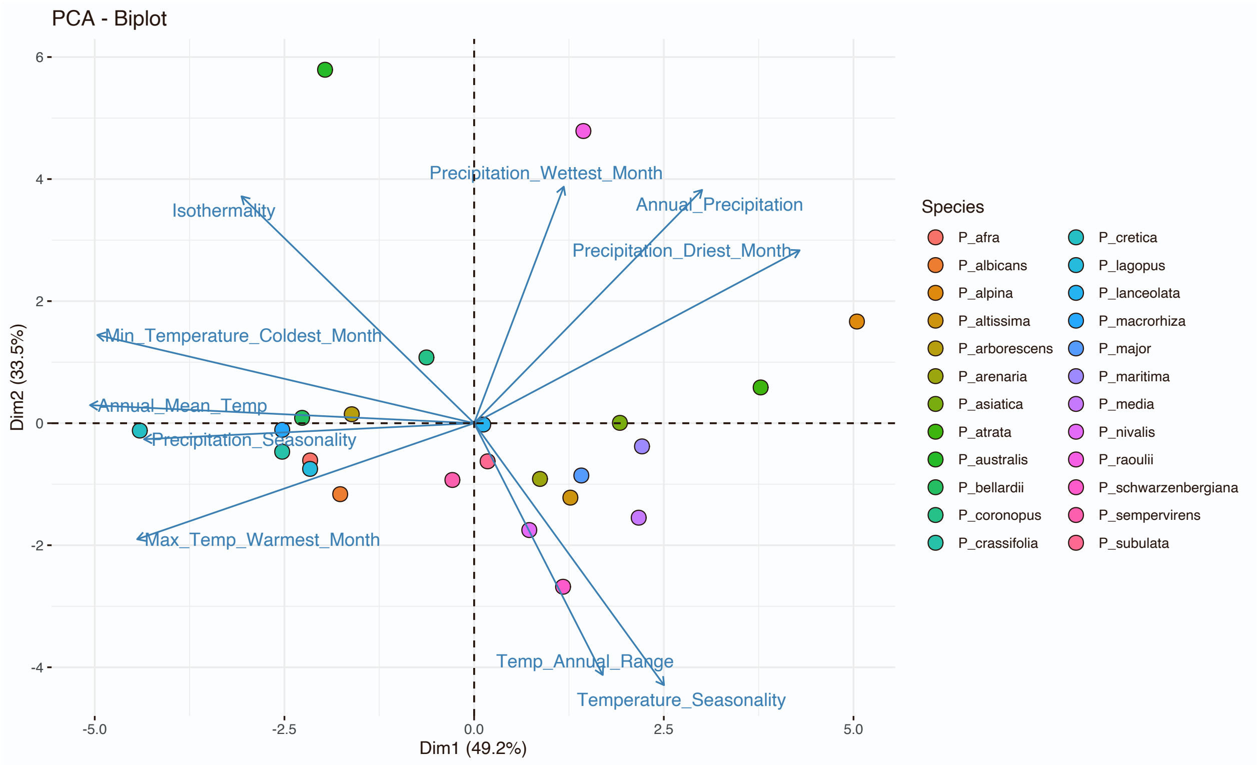

Figure S2. Principal Component Analysis (PCA) of *Plantago* species based on 10 bioclimatic variables representing the average climatic niche of each species, **related to STAR methods**.

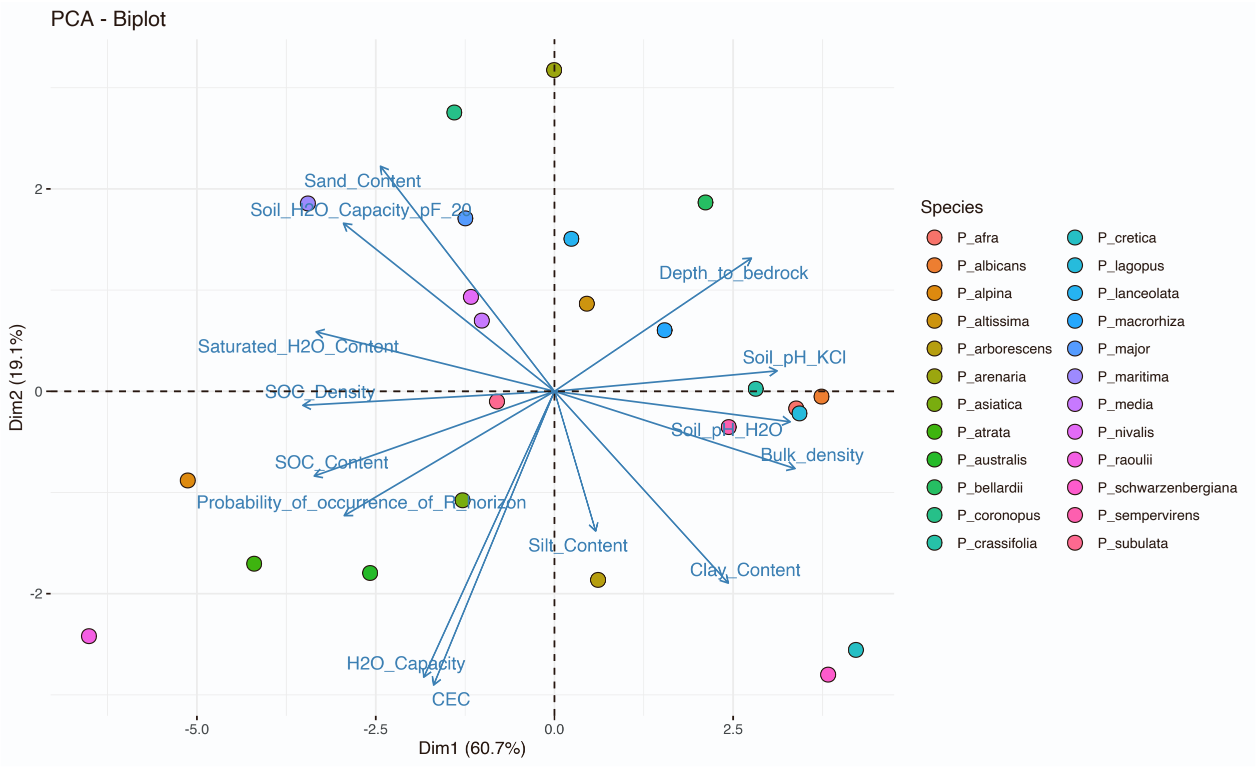

Figure S3. Principal Component Analysis (PCA) of *Plantago* species based on 14 soil physico-chemical properties, representing the pedological niche of each species, **related to STAR methods**.

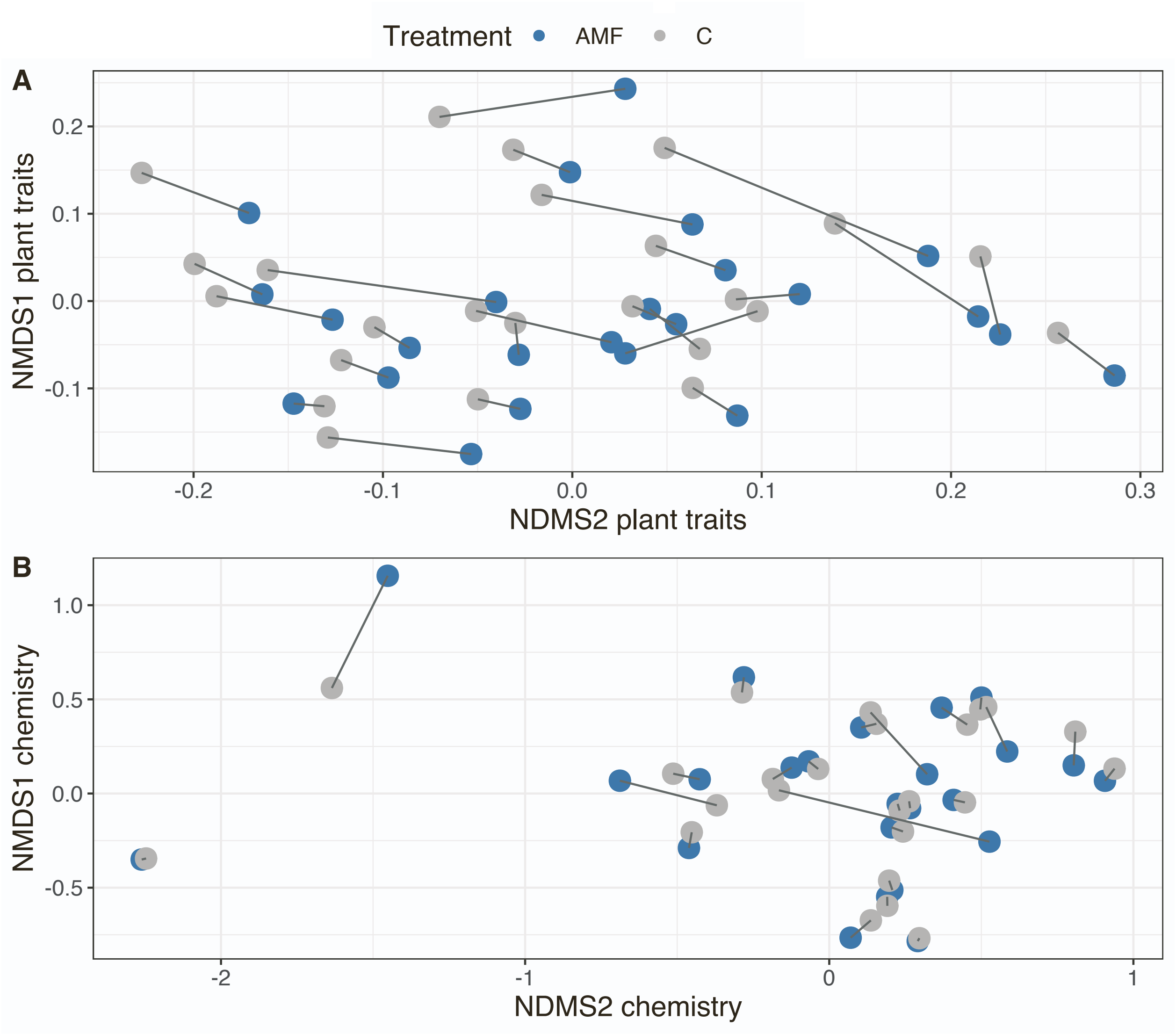

Figure S4. Multivariate distance of plant traits across AMF treatments, **related to STAR methods**, results section 4 and Figure 3. Shown are non-metric multidimensional scaling (NDMS) plots for A) the plant trait matrix, and B) the chemical trait (iridoid glycosides (IGs) matrix across 24 *Plantago* species. Grey lines connect the same species that grew with (blue dots) or without (grey dots) arbuscular mycorrhizal fungi (AMF).

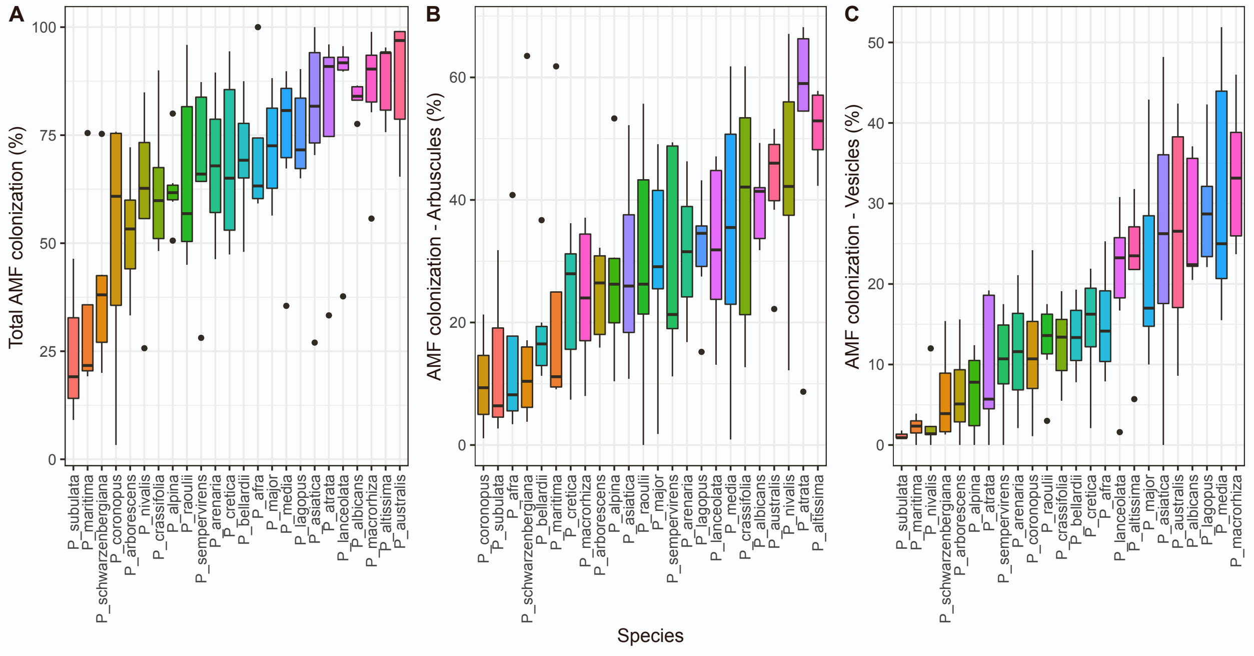

Figure S5. Mycorrhization levels across *Plantago* species, **related to Figure 1A**. Boxplots in panels **A**, **B** and **C** show the median and extremes in variation on total arbuscular mycorrhizal colonization intensity, AMF- arbuscule colonization intensity, and AMF-vesicles colonization intensity in roots (percent) across 24 *Plantago* species surveyed in this study, respectively. The gradient of colour represents the variation in AMF colonization from low values to high value of colonization, and species in panel **B** and **C** are color-coded as in panel **A**.

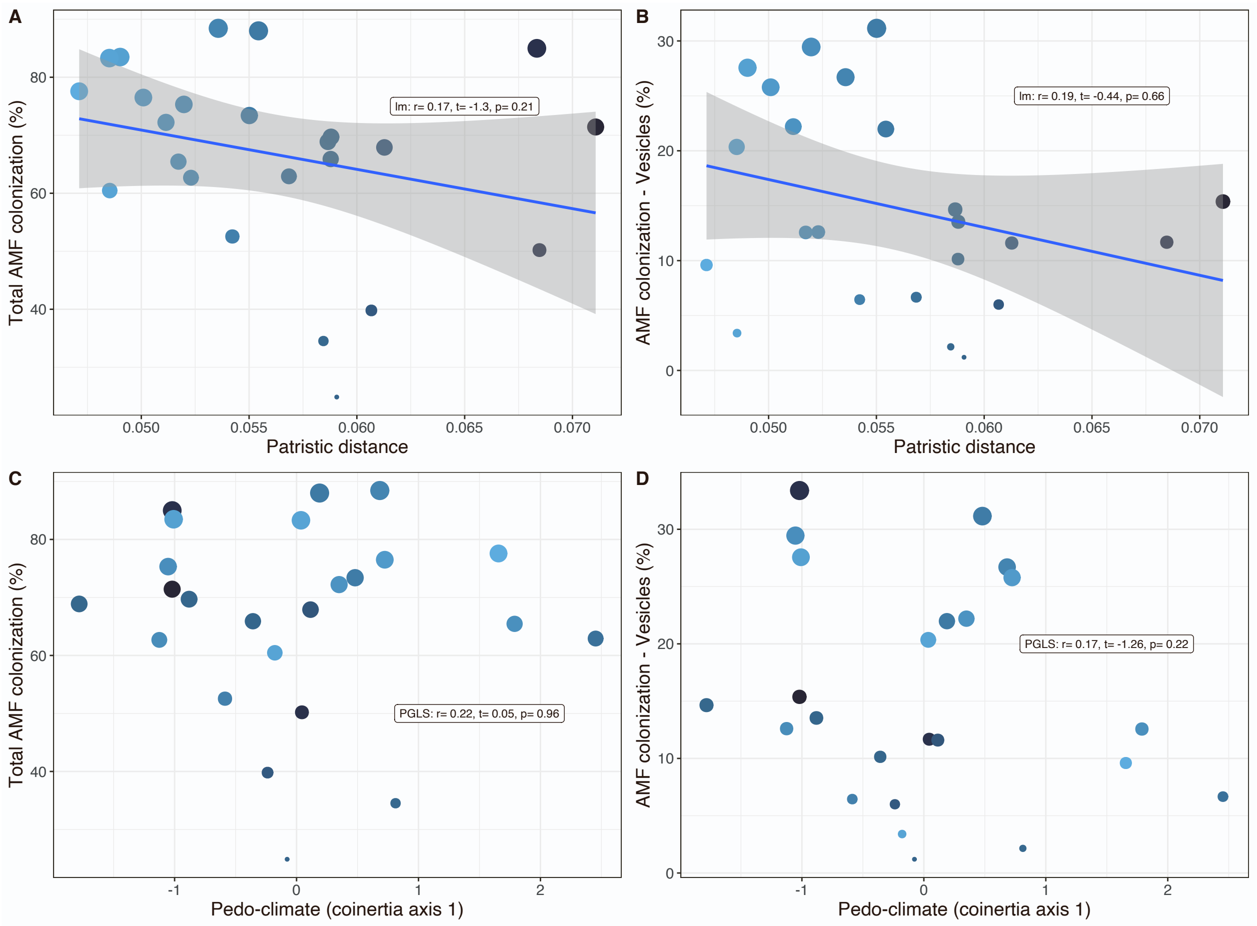

Figure S6. Phylogenetic pattern in mycorrhizal colonization across *Plantago* species, **related to Results section 1**. Panels **A**) and **C)** represent the correlation between the total AMF colonization intensity (arbuscules + vesicles + hyphae) with the phylogenetic branch length, and the pedo-climatic niche of all species, respectively. Panels **B**) and **D**) represent the correlation between the AMF-vesicles (alone) colonization intensity with the phylogenetic branch length, and the pedo-climatic niche of all species, respectively. Dots are coded based on the root-to-tip distance of each species, with lighter colours indicating shorter branch length. The grey-shaded area around the prediction line (blue) represents 95% confidence level interval for predictions from a linear model.

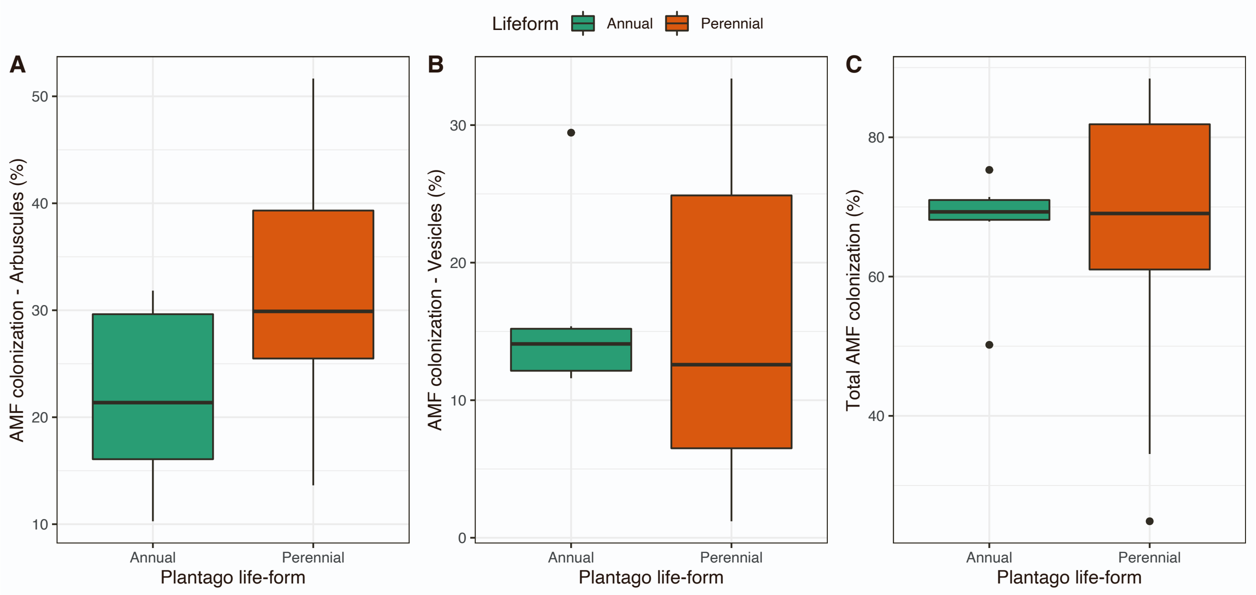

Figure S7. *Plantago* species lifeform effect on arbuscular mycorrhizal fungi colonization, **related to the Discussion section**. **A**) colonization by arbuscules, **B**) colonization by vesicles, and **C**) all AMF structures. Green boxplots represent average values for all annual and biannual species, (n = 6) and brown boxplots include all perennial species (n = 18). For statistical differences among lifeforms see Table S5.
